# High Cytotoxic Efficiency of Lentivirally and Alpharetrovirally Engineered CD19-Specific Chimeric Antigen Receptor Natural Killer Cells Against Acute Lymphoblastic Leukemia

**DOI:** 10.3389/fimmu.2019.03123

**Published:** 2020-01-24

**Authors:** Stephan Müller, Tobias Bexte, Veronika Gebel, Franziska Kalensee, Eva Stolzenberg, Jessica Hartmann, Ulrike Koehl, Axel Schambach, Winfried S. Wels, Ute Modlich, Evelyn Ullrich

**Affiliations:** ^1^Experimental Immunology, Department for Children and Adolescents Medicine, University Hospital Frankfurt, Goethe University, Frankfurt am Main, Germany; ^2^Division of Pediatric Stem Cell Transplantation and Immunology, Department for Children and Adolescents Medicine, University Hospital Frankfurt, Goethe University, Frankfurt am Main, Germany; ^3^German Cancer Consortium (DKTK) Partner Site Frankfurt/Mainz, Frankfurt am Main, Germany; ^4^Division of Medical Biotechnology, Paul-Ehrlich-Institut, Langen, Germany; ^5^Fraunhofer Institute for Cell Therapy and Immunology (IZI), Leipzig, Germany; ^6^Institute of Cellular Therapeutics, Hannover Medical School, Hanover, Germany; ^7^Institute of Clinical Immunology, Faculty of Medicine, University Leipzig, Leipzig, Germany; ^8^Institute of Experimental Hematology, Hannover Medical School, Hanover, Germany; ^9^Division of Hematology/Oncology, Boston Children's Hospital, Harvard Medical School, Boston, MA, United States; ^10^Georg-Speyer-Haus, Institute for Tumor Biology and Experimental Therapy, Frankfurt am Main, Germany; ^11^Frankfurt Cancer Institute, Goethe University, Frankfurt am Main, Germany; ^12^Research Group for Gene Modification in Stem Cells, Division of Veterinary Medicine, Paul-Ehrlich Institute, Langen, Germany

**Keywords:** chimeric antigen receptor, natural killer cells, acute lymphoblastic leukemia, alpharetroviral vector, lentiviral vector, gene therapy, CD19

## Abstract

Autologous chimeric antigen receptor-modified (CAR) T cells with specificity for CD19 showed potent antitumor efficacy in clinical trials against relapsed and refractory B-cell acute lymphoblastic leukemia (B-ALL). Contrary to T cells, natural killer (NK) cells kill their targets in a non-antigen-specific manner and do not carry the risk of inducing graft vs. host disease (GvHD), allowing application of donor-derived cells in an allogenic setting. Hence, unlike autologous CAR-T cells, therapeutic CD19-CAR-NK cells can be generated as an off-the-shelf product from healthy donors. Nevertheless, genetic engineering of peripheral blood (PB) derived NK cells remains challenging and optimized protocols are needed. In our study, we aimed to optimize the generation of CD19-CAR-NK cells by retroviral transduction to improve the high antileukemic capacity of NK cells. We compared two different retroviral vector platforms, the lentiviral and alpharetroviral, both in combination with two different transduction enhancers (Retronectin and Vectofusin-1). We further explored different NK cell isolation techniques (NK cell enrichment and CD3/CD19 depletion) to identify the most efficacious methods for genetic engineering of NK cells. Our results demonstrated that transduction of NK cells with RD114-TR pseudotyped retroviral vectors, in combination with Vectofusin-1 was the most efficient method to generate CD19-CAR-NK cells. Retronectin was potent in enhancing lentiviral/VSV-G gene delivery to NK cells but not alpharetroviral/RD114-TR. Furthermore, the Vectofusin-based transduction of NK cells with CD19-CARs delivered by alpharetroviral/RD114-TR and lentiviral/RD114-TR vectors outperformed lentiviral/VSV-G vectors. The final generated CD19-CAR-NK cells displayed superior cytotoxic activity against CD19-expressing target cells when compared to non-transduced NK cells achieving up to 90% specific killing activity. In summary, our findings present the use of RD114-TR pseudotyped retroviral particles in combination with Vectofusin-1 as a successful strategy to genetically modify PB-derived NK cells to achieve highly cytotoxic CD19-CAR-NK cells at high yield.

## Introduction

Treatment of refractory and relapsed B-cell acute lymphoblastic leukemia (B-ALL) is challenging and relies on therapies such as chemotherapy, monoclonal antibodies, and hematopoietic stem cell transplantation (1). Nevertheless, the outcome of these patients remains poor (2, 3). In clinical trials, autologous chimeric antigen receptor-modified (CAR) T cells with specificity for CD19 showed potent antitumor efficacy against relapsed and refractory B-ALL (4–8). However, the usage of CAR-T cells is restricted to an autologous setting as allogenic T cells, even if HLA-matched, carry the risk of inducing graft vs. host disease (GvHD) (9, 10). Additionally, generation of relevant doses of CAR-T cells for heavily pretreated patients suffering from lymphopenia may be impracticable. Hence, an allogenic product for those patients might offer a suitable alternative.

Natural killer (NK) cells are cytolytic lymphocytes that represent the first line of defense against aberrant cells caused by viral infections or malignancies (11, 12). Contrary to T cells, they do not cause GvHD, allowing application of donor-derived cells in an allogenic setting, which has been successfully demonstrated with cytokine expanded donor-derived NK cells (13–18). Therefore, CAR-NK cells generated as an off-the-shelf product from healthy donors, offer an alternative to CAR-T cells. Additionally, NK cells kill their targets in a non-antigen-specific manner using germ-line encoded receptors which recognize and kill aberrant cells (19, 20). Thereby, NK cells detect aberrant cells, which express elevated levels of stress induced ligands (“induced self”) (21, 22), or cells, which downregulate or lose their human leukocytes antigen (HLA) I molecules (“missing self”) (23, 24), a mechanism to escape T cell mediated killing (25). Furthermore, most NK cells express CD16 (FcγRIII), which allows them to mediate antibody-dependent cell-mediated cytotoxicity (ADCC) (11). Taken together, CD19-CAR-NK cells might attack tumor cells utilizing both the CAR-dependent and CAR-independent NK-cell intrinsic mechanisms, potentially killing a heterogeneous tumor cell population.

NK cells for genetic modification can be derived from different sources. The NK cell line NK-92 is widely used for CAR-NK cell engineering (26) and expressing CD19-specific CARs, they already showed high cytotoxic activity against leukemic cells (27). Nevertheless, as NK-92 cells are tumor derived cells (28), they must be irradiated prior clinical use to avoid permanent engraftment in patients. Additionally, they do not express CD16 and thus are unable to mediate ADCC (28, 29). Another possible source for CAR-NK cell engineering are NK cells enriched from peripheral blood (PB) (30). Compared to NK cell lines, they express a wider range of activating receptors (31) and do not require irradiation, making them a favorable NK cell source. First clinical trials using CAR-NK cells derived from a variety of sources (including PB-derived NK cells) have been started (32, 33).

To date, genetic engineering of PB-derived NK cells remains challenging, mainly as gene transfer efficiency into NK cells is low. Mostly retroviral vectors are used to genetically modify NK cells and a variety of protocols including different viral vector systems (alpharetroviral, gammaretroviral, or lentiviral), culture conditions and transduction enhancers are described [for review see (34)]. Often, genetically modified K562 cells, a tumor derived cell line, are used as feeder cells to enhance expansion [for review see (35)] and transduction of NK cells (36, 37). Previous approaches generated CD19-CAR-NK cells from PB-derived NK cells containing variable levels of CAR^+^ cells depending on the used protocol. In a feeder cell-based protocol a high number of transduced cells was achieved (36). In contrast, PB-derived NK cells cultured and transduced in a feeder cell-free protocol underperformed, reaching only low amounts of transduced cells (38). Hence, optimized feeder cell-free protocols achieving transduced NK cells at high yield are lacking.

In our study, we focused on optimizing the generation of PB-derived CD19-CAR-NK cells by viral transduction using a feeder cell-free culture. In addition to earlier studies, we compared lentiviral and alpharetroviral transduction, different transduction enhancers (Retronectin and Vectofusin-1) and NK cell isolation methods (NK cell enrichment and CD3/CD19 depletion) to identify the most efficient methods for genetic engineering of NK cells. Finally, we generated CD19-CAR-NK cells at high yield with an increased cell killing activity of leukemic cells.

## Materials and Methods

### Cell Lines

Human embryonic kidney (HEK) 293T cells and human fibrosarcoma cell line HT1080 were cultured in Dulbecco's Modified Eagle Medium (DMEM) supplemented with 10% fetal bovine serum (FBS) and 1% L-glutamine (all Gibco). The cells were split every 2–3 days. Chronic myologenous erythroleukemia cell line K562 was cultured in Roswell Park Memorial Institute (RPMI) 1640 medium, GlutaMAX (Gibco) containing 10% FBS and 1% penicillin/streptomycin (Pen/Strep; Gibco). ALL cell line Sup-B15 was cultured in RPMI 1640 medium, GlutaMAX supplemented with 15% FBS and 1% Pen/Strep. Both leukemic cell lines were split every 3–4 days.

### Isolation of NK Cells

This study was approved by the Ethics Committee of the Goethe University Frankfurt, Germany (approval no. 329/10). All participants gave written informed consent in accordance with the Declaration of Helsinki. NK cells were isolated from freshly generated, healthy and anonymous donor buffy coats provided by the German Red Cross Blood Donation Service (DRK-Blutspendedienst Baden-Württemberg-Hessen, Frankfurt, Germany) using Ficoll density gradient centrifugation (Biocoll, Biochrom) to isolate peripheral blood mononuclear cells (PBMCs) and finally enrich NK cells by immunomagnetic negative selection. Enrichment of NK cells were performed either using EasySep Human NK cell Enrichment Kit (in the following referred to as NK cell enrichment) or a combination of using EasySep Human CD3 Positive Selection Kit II and EasySep Human CD19 Positive Selection Kit II (in the following referred to as CD3/CD19 depletion) according to manufacturer's instructions (all StemCell Technologies). The CD3/CD19 depletion uses positive selection of T cells and B cells to deplete them from unbound PBMCs mainly CD56^+^CD3^−^ NK cells and other remaining lymphocytes (mainly CD14^+^ dendritic cells, monocytes etc.). NK cell purity and characterization were determined by flow cytometry using fluorochrome-conjugated antibodies against CD56 BV421 (clone NCAM16.2), CD3 BUV395 (clone SK7), CD19 BB515 (clone HIB19), CD20 BUV737 (clone 2H7), CD45 BV510 (clone HI30) (all BD Biosciences), CD14 BV711 (clone M5E2), and CD16 PE (clone 3G8) (both Biolegend). Detailed gating strategy is shown in the Supplementary Figure 1. Freshly isolated cells were cultured in X-VIVO 10 medium (Lonza) supplemented with 5% heat-inactivated human plasma (DRK-Blutspendedienst), 1% Pen/Strep and 10 ng/ml IL-15 (Peprotech) at a concentration of 2 × 10^6^ cells/ml (NK cell enrichment) or 3 × 10^6^ cells/ml (CD3/CD19 depletion).

### Retroviral SIN Vectors

The lentiviral EGFP vector (RRL.PPT.SF.GFPpre), the alpharetroviral EGFP vector (AlphaSIN.noTATA) and the lentiviral CD19-CAR vector (S-CD19-CAR-W) have been previously described (27, 39–42). Briefly, the vectors were equipped with a Spleen Focus-Forming Virus (SFFV) promotor to drive expression of the transgene cassettes (EGFP, second-generation CD19-CAR). The second-generation CD19-CAR consists of an immunoglobulin heavy-chain signal peptide (SP), the anti-CD19 single-chain variable fragment (scFv) domain of a murine monoclonal antibody (FMC63) (43), a myc-tag (Myc), a CD8α hinge region, the CD28 transmembrane domain, and a composite CD28-CD3ζ signaling domain (27, 41). The alpharetroviral CD19-CAR vector (Alpha-CD19-CAR) was generated by removing the EGFP transgene of the alpharetroviral EGFP vector and replacing it by the CD19-CAR transgene of the lentiviral CD19-CAR vector. More detailed architectures of vectors are described in the Supplementary Figure 2.

### Production of Retroviral Vector Particles and Titer Estimation

Viral vector particles were produced by transfection of HEK 293T cells using the calcium phosphate precipitation method. Twenty-four hours prior to transfection 4–5 × 10^6^ or 10–11 × 10^6^ HEK 293T cells were seeded on a 10 or 15 cm culture dish in DMEM supplemented with 10% FBS and 1% L-glutamine (all Gibco). For transfection, the medium was exchanged with DMEM containing 10% FBS, 1% L-glutamine, 1% Pen/Strep, and 25 μM chloroquine (Sigma-Aldrich). To produce lentiviral particles, following amounts of plasmids were added: 10 or 23 μg of the transfer vector (RRL.PPT.SF.GFPpre or S-CD19-CAR-W), 10 or 23 μg of a lentiviral gag/pol plasmid (pcDNA3 g/p 4xCTE) (39), 5 or 11.5 μg of a Rev plasmid (pRSV-Rev) and 1.5 μg or 3.45 μg of VSV-G (pMD2.G) envelope plasmid or 4.5 μg or 9.2 μg of RD114-TR (44) envelope plasmid. For production of alpharetroviral particles 5 or 11.5 μg of the transfer vector (AlphaSIN.noTATA or Alpha-CD19-CAR), 2.5 or 5.75 μg of an alpharetroviral gag/pol plasmid (pcDNA3.ASLV gp co) (40) and 2 or 9.2 μg of RD114-TR envelope plasmid were added. Medium was changed after 12–18 h. Supernatants containing the viral particles were collected 24, 36, and 48 h after transfection. They were filtered through a 0.22 μm filter, concentrated by centrifugation at 50,000 × g for 1 h (VSV-G, RD114-TR) or overnight at 4,500–11,600 × g (RD114-TR), resuspended in X-VIVO 10 medium and stored at −80°C. To enhance purification of viral particles some supernatants were centrifuged with the addition of 20% Sucrose/PBS. Viral titers were estimated by transducing HT1080 cells with different volumes of viral supernatant. 50,000 HT1080 cells per well of 12-well plates were seeded. After adherence, serial dilutions of viral supernatant and 4 μg/ml protamine sulfate were added. Three days later the percentage of EGFP^+^ or CD19-CAR^+^ cells was quantified by flow cytometry. Titers were calculated using following formula: Titer (viral particles/ml) = 50,000 cells × transgene positive events (%)/volume of supernatant (ml).

### Transduction of Primary Human NK Cells

On day four after isolation, NK cells were transduced with lentiviral and alpharetroviral vector particles. Prior to transduction, NK cell purity (percent amount of CD56^+^CD3^−^ cells) was determined by flow cytometry as described above. NK cell purity was 90.0% for NK cells isolated by NK cell enrichment and 90.9% for NK cells isolated by CD3/CD19 depletion. For Retronectin-mediated transduction, flat bottomed 96-well plates were pre-coated with 25 μg/ml Retronectin (Takara) for at least 2 h at room temperature or overnight at 4°C. In the next step, Retronectin was removed and wells were blocked for 30 min with PBS containing 2% BSA. The blocking solution was discarded, and wells were washed once with HBSS/HEPES. Viral supernatants were added onto the Retronectin coated wells and the plate was centrifuged at 1,000 × g for 30 min at 4°C. Afterwards, NK cells were added reaching cell densities of 2 × 10^6^ cells/ml for transductions with EGFP vectors or 10^5^ cells/ml for transductions with CD19-CAR vectors. Finally, 10 ng/ml IL-15 was added. For Vectofusin-1 (Miltenyi Biotech) based transduction, Vectofusin-1 and viral supernatant were diluted separately in X-VIVO 10 media supplemented with 5% heat-inactivated human plasma and 1% Pen/Strep. Both diluted solutions contained identical volumes, were finally mixed, shortly vortexed and incubated for 5–10 min. Next, the mixture was added to NK cells plated on flat bottomed 96-well plates reaching cell densities of 2 × 10^6^ cells/ml for transductions with EGFP vectors or 10^5^ cells/ml for transductions with CD19-CAR vectors. The final concentration of Vectofusin-1 was 10 μg/ml per well. Finally, 10 ng/ml IL-15 was added and the plate was centrifuged at 800 × g for 1.5 h at 32°C. Both protocols contained a change of medium 24 h post-transduction. Half of the medium was discarded and replaced by fresh medium containing 10 ng/ml IL-15. Non-transduced (NT) NK cells of the same donor were used as negative controls. The NT-NK cells were manipulated the exact same way as the transduced (either EGFP or CAR) NK cells except no pseudotyped viral particles were added.

### Flow Cytometric Analysis of Transduced NK Cells

Transgene expression, CD16 distribution and contamination with CD3 and CD14 positive cells of gene modified NK cells were analyzed 3 days after transduction by using a BD FACSCelesta flow cytometer (BD Biosciences). Fluorochrome-conjugated antibodies against CD56 BV786 (clone NCAM16.2), CD16 PE-CF 594 (clone 3G8), CD3 BUV395 (clone Sk7) (all BD Biosciences) and CD14 BV711 (Biolegend) were used. CD19-CAR expression was confirmed with a PE conjugated Myc-tag-specific antibody (clone 9B11, Cell Signaling Technology). NT-NK cells of the same donor were used as negative controls. Data were analyzed using FlowJo (FlowJo LLC). Detailed gating strategy is shown in the Supplementary Figure 3.

### CD19 Expression and Cytotoxicity Assay

CD19 expression on the surface of K562 and Sup-B15 cells was analyzed by flow cytometry using a fluorochrome-conjugated antibody against CD19 BB515 (clone HIB19; BD Biosciences). Cytotoxicity of NK cells against K562 and Sup-B15 cells was determined in a flow cytometry-based cytotoxicity assay. Assays were performed with CD19-CAR-NK cells and NT-NK cells of the same donors 3 days after transduction. Target cells were harvested and stained with Cell Trace CFSE proliferation kit (Invitrogen) in a final concentration of 5 μM. Afterwards, they were washed with PBS and resuspended in X-VIVO 10 media supplemented with 5% heat-inactivated human plasma and 1% Pen/Strep. Target cells and NK cells were combined in U-bottom shaped 96-well plates at effector to target (E:T) ratios of 1:1, 0.5:1, 0.25:1, and 0.1:1 for 4 h, using 25,000 target cells per well in a total volume of 100 μl. After a coincubation time of 4 h plates were centrifuged at 300 × g for 5 min, the supernatant was removed, and cells were finally harvested and resuspended in 400 μl DAPI solution (DAPI 1 mg/ml, diluted in PBS at 1:6,000) (AppliChem GmbH). From each well the same amount of target cells was acquired using a BD FACSCelesta flow cytometer (BD Biosciences). Dead target cells were identified as CFSE and DAPI double positive and samples of target cells only were used as controls for spontaneous cell lysis. Detailed gating strategy is shown in the Supplementary Figures 5, 6.

### Cytometric Bead Array

Cytokine release by NT-NK and CD19-CAR-NK cells was examined using BD Cytometric Bead Array (CBA) analyses (BD Bioscience). Supernatants of NT-NK cells, CD19-CAR-NK cells, and Sup-B15 cells in culture as well as after coculture in cytotoxicity assays (E:T ratio of 1:1) were frozen at −80°C. Cytokine concentrations in supernatants were measured using BD CBA Flex Sets for granulocyte-macrophage colony-stimulating factor (GM-CSF), tumor necrosis factor (TNF)-α; macrophage inflammatory protein (MIP)-1α and interferon (IFN)-γ (BD Biosciences). The tests were performed according to the manufacturer's instructions. Data were acquired with the BD FACSVerse Bioanalyzer and were quantitated using the FCAP Array software (v3.0.1; BD Biosciences).

### Long Term Culture of NK Cells

For long term culture of CD19-CAR-NK and NT-NK cells, NK cells were cultured in NK MACS medium (Miltenyi Biotec) supplemented with 5% heat-inactivated human plasma, 1% Pen/Strep, and 10 ng/ml IL-15 at a starting concentration of 1 × 10^6^ cells/ml on the day of transduction (day 0). On day 3, 7, 11, and 14 post-transduction, transduction efficiency was determined by flow cytometry. On these days, NK cells were additionally counted, and the cell number was adjusted to 2 × 10^6^ cells/ml in fresh NK MACS medium (5% heat-inactivated human plasma, 1% Pen/Strep, and 10 ng/ml IL-15) for expansion analysis.

### Statistical Analysis

For statistical analysis, under the assumption of normal distribution of the NK function in healthy donors, data were analyzed by two-tailed paired *t*-test. The resulting approximatively *p* < 0.05 were considered significant and are indicated in the results. Only data from experiments with three or more donors (*n* ≥ *3*) were considered for statistical analysis. Statistical calculations were performed with GraphPad PRISM version 8 (GraphPad Software, Inc.).

## Results

### Vectofusin-1 Promotes Lentiviral Transduction of NK Cells as Efficient as Retronectin

**Figure 1:**
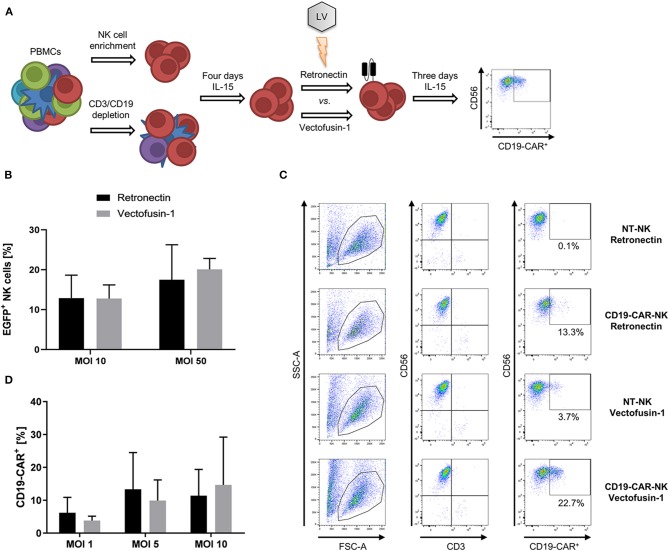
Comparative lentiviral transduction of NK cells using Retronectin and Vectofusin-1. **(A)** Scheme of the transduction procedure. NK cells were isolated from PBMCs either by NK cell enrichment or CD3/CD19 depletion and cultivated for 4 days with IL-15 prior to transduction comparing the enhancers Retronectin and Vectofusin-1. After transduction, NK cells were cultivated for 3 days with IL-15 until transduction efficiency was analyzed by flow cytometry. **(B)** NK cells from three donors (*n* = 3) were transduced with VSV-G pseudotyped lentiviral EGFP particles at two different multiplicities of infection (MOI) and with two different transduction enhancers. **(C)** Gating strategy to estimate the transduction efficiency of NK cells transduced with VSV-G pseudotyped lentiviral CD19-CAR particles (e.g., for more detailed gating strategy see Supplementary Material). NK cells were identified as CD56^+^CD3^−^ leukocytes (first and second column). From those CD19-CAR^+^ NK cells were estimated (third column). In the first and second row representative data of NK cells are depicted that were transduced with Retronectin at MOI 5 vs. non-transduced (NT) NK cells from NK cell preparations of the same donor. In the third and fourth row data from NK cells transduced with Vectofusin-1 at MOI 5 vs. NT-NK cells are shown. Percentage of false positive CD19-CAR events in NT-NK cells was subtracted from the percentages measured in the belonging transduced NK cells. Shown are the dot plots of one donor. **(D)** NK cells from four donors (*n* = 4) were transduced with VSV-G pseudotyped lentiviral CD19-CAR particles at shown MOIs and with two different transduction enhancers. Shown are mean values + SD. Statistical analysis was performed using two-tailed student's paired *t*-test. No significant differences were observed between analyzed groups in **(B,D)** as *p*-values were >0.05.

To identify the most efficacious method for genetic engineering of NK cells, we first focused on optimizing transduction of NK cells with lentiviral vectors. For that purpose, NK cells isolated from PBMCs by NK cell enrichment or CD3/CD19 depletion were pre-activated for 4 days with low dose IL-15 (10 ng/ml) prior to lentiviral transduction comparing two transduction enhancers, afterwards the gene modified NK cells were continuously cultured in IL-15 for at least 3 days when finally phenotyping was performed (Figure 1A). NK cells isolated by NK cell enrichment were transduced with the VSV-G pseudotyped lentiviral EGFP vector comparing Retronectin and Vectofusin-1 for enhancement (Figure 1B). Overall transduction rates did not significantly differ between both protocols. Mean transduction rates of 12.9% with Retronectin and 12.8% with Vectofusin-1 were reached at MOI 10. A higher MOI of 50 could increase the rates up to 17.5% with Retronectin and 20.1% with Vectofusin-1. Next, we addressed generation of CD19-CAR-NK cells out of NK cells isolated by NK cell enrichment using the lentiviral second-generation CD19-CAR (lentiviral/VSV-G CD19-CAR) and compared the two transduction enhancers (Figures 1C,D). We could confirm the equality of Vectofusin-1 and Retronectin in their ability to enhance lentiviral gene delivery using the CD19-CAR. Transduction efficiencies of NK cells highly varied between different donors and, taken all MOIs together, a span of 0–34.0% transduced cells was reached. Using higher MOIs of 20 and 50 did not improve transduction efficiencies and a decline in total viable cells was observed with these MOIs (data not shown), so that the maximum used MOI was 10. The highest rates of transduced cells reached with Retronectin were 28.7% (MOI 5) and with Vectofusin-1 34.0% (MOI 10). Comparing mean transduction efficiencies, the best condition with the highest transduction rates was Vectofusin-1 combined with MOI 10 reaching 14.7% CD19-CAR-NK cells. Overall there was no significant difference between Retronectin or Vectofusin-1 based lentiviral transduction to generate CD19-CAR-NK cells.

### Different Isolation Methods Are Suitable for the Generation of CD19-CAR-NK Cells

**Figure 2:**
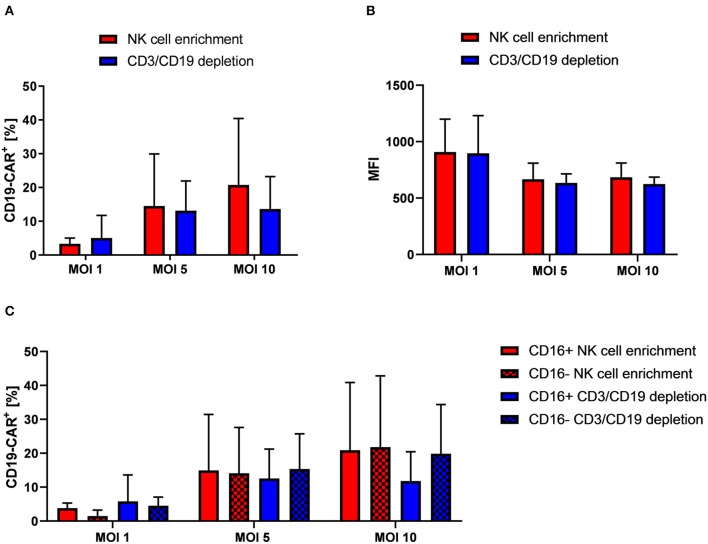
NK cells isolated from PBMCs by NK cell enrichment or CD3/CD19 depletion show similar transduction efficiencies when transduced with VSV-G pseudotyped lentiviral CD19-CAR particles. **(A)** Vectofusin-1 based transduction of differently isolated NK cells was performed with VSV-G pseudotyped lentiviral CD19-CAR particles at shown MOIs. NK cells from four different donors were used *(n* = *4)*. **(B)** Mean fluorescence intensities (MFI) of CD19-CAR in transduced cells. Data show average MFIs of CD19-CAR^+^ cells transduced with depicted MOIs as shown in **(A)**. **(C)** CD19-CAR expression of CD16^+^ and CD16^−^ NK cell subpopulations. CD19-CAR expression of CD16^+^ and CD16^−^ NK cell subpopulations of transduced cells depicted in **(A)** are shown *(n* = *4)*. Shown are mean values + SD. Statistical analysis was performed using two-tailed student's paired *t-*test. No significant differences were observed between analyzed groups transduced with the same MOI as *p*-values were >0.05.

After we had established Vectofusin-1 based transduction of NK cells with VSV-G pseudotyped lentiviral vectors, we used this procedure in further lentiviral transduction experiments. Reason for this was the shorter and simpler usage of Vectofusin-1 compared to Retronectin. In the next step, we addressed whether NK cells isolated by CD3/CD19 depletion for genetic engineering may be more suitable for transduction with VSV-G pseudotyped second generation CD19-CAR lentiviral vectors as remaining bystander cells might support successful transduction. For that, NK cells isolated by different protocols from the same donors were compared. Overall, no significant differences on transduction efficiency could be observed between both isolation methods (Figure 2A, a representative dot plot is shown in Supplementary Figure 4). At MOI 10 highest rates of CD19-CAR-NK cells were reached for both isolation methods: 13.7% CD19-CAR-NK cells for CD3/CD19 depleted NK cells and 20.8% CD19-CAR-NK cells for NK cells isolated by NK cell enrichment. Mean fluorescence intensities (MFIs) of CD19-CAR-NK cells were on a same level for both isolation methods, regardless of used MOI (Figure 2B). Interestingly, highest MFIs for both isolation methods were reached with MOI 1. Furthermore, we analyzed CD19-CAR expression of NK cell subpopulations. Subpopulations are defined as CD56^high^CD16^−^ (hereinafter: CD16^−^) and cytotoxic CD56^dim^CD16^+^ NK cells (hereinafter: CD16^+^). In general, NK cell subpopulation distribution was not influenced by transduction (Supplementary Figure 7). Remarkably, CD19-CAR expression of CD16^+^ and CD16^−^ NK cell subpopulations did not significantly differ when compared within one isolation method, nor when one subgroup from one isolation method was compared with the belonging subgroup from the other isolation method (Figure 2C). Overall the relative CD19-CAR expression of the CD16^+^ and CD16^−^ NK cell subpopulations matched with the CD19-CAR expression of the total NK cell population (Figures 2A,C).

### Alpharetroviral/RD114-TR Engineering of CD19-CAR-NK Cells Outperforms Lentiviral/VSV-G Engineering of CD19-CAR-NK Cells

**Figure 3:**
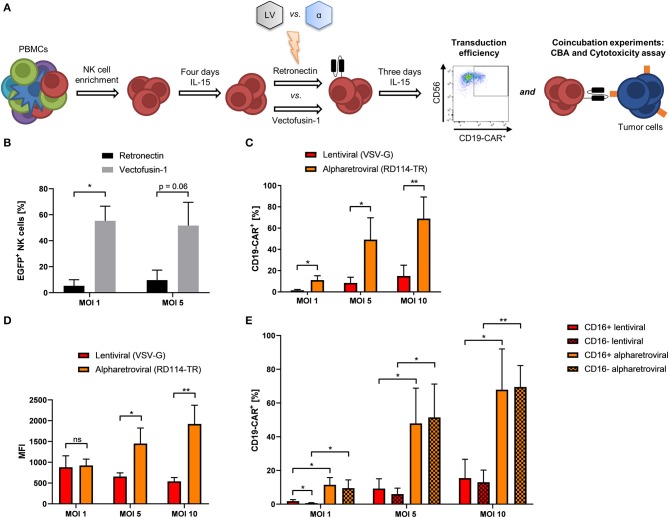
Transduction rates of alpharetrovirally/RD114-TR modified NK cells outperform those of lentivirally/VSV-G modified NK cells. **(A)** Scheme of the comparative transduction procedure. NK cells were isolated from PBMCs by NK cell enrichment and cultivated for 4 days under the influence of IL-15 prior to transduction comparing the enhancers Retronectin and Vectofusin-1 and the lentiviral (LV) and alpharetroviral (α) vector system. After transduction, NK cells were cultivated for 3 days with IL-15 until transduction efficiency was analyzed by flow cytometry and functional assays were performed. **(B)** NK cells from three donors *(n* = *3)* were transduced with RD114-TR pseudotyped alpharetroviral EGFP particles at shown MOIs. **(C)** Vectofusin-1 mediated transduction of NK cells from four donors *(n* = *4)* was performed with RD114-TR pseudotyped alpharetroviral CD19-CAR particles or VSV-G pseudotyped lentiviral CD19-CAR particles at different MOIs. **(D)** MFI of CD19-CAR in transduced cells. Data show average MFIs of CD19-CAR^+^ cells transduced with depicted MOIs as shown in **(B)**. **(E)** CD19-CAR expression of CD16^+^ and CD16^−^ NK cell subpopulations. CD19-CAR expression of CD16^+^ and CD16^−^ NK cell subpopulations of transduced cells depicted in **(B)** are shown *(n* = *4)*. Shown are mean values + SD. Statistical analysis was performed using two-tailed student's paired *t*-test. **p < 0.01; *p < 0.05; ns, not significant.

Even though CD19-CAR-NK cells could be generated using lentiviral transduction, the resulting efficiencies remained on a moderate level independent of the used protocols. To further improve transduction efficiencies of NK cells to obtain genetically modified NK cells at high yield, we compared not only transduction efficiency, but also function of lentiviral and alpharetroviral CD19-CAR-vectors (Figure 3A). Previous work could show the feasibility of the alpharetroviral system for the genetic engineering of NK cells (45). For our study, alpharetroviral vectors were pseudotyped with RD114-TR because higher functional infectivity could be achieved with this envelope (own unpublished data) (40, 45). First, NK cells were transduced with the alpharetroviral EGFP vector comparing Retronectin and Vectofusin-1 based transduction. Interestingly, Vectofusin-1 promoted alpharetroviral transduction of NK cells better than Retronectin, reaching up to 70% transduced cells (Figure 3B). Next, we generated a CD19-CAR alpharetroviral vector by substituting the EGFP transgene cassette of the alpharetroviral EGFP vector for the second-generation CD19-CAR cassette of the lentiviral CD19-CAR vector. Vectofusin-1 based transduction of NK cells was performed using the new alpharetroviral CD19-CAR vector (alpharetroviral/RD114-TR CD19-CAR) and the lentiviral/VSV-G CD19-CAR vector. Across all MOIs the alpharetroviral/RD114-TR CD19-CAR vector outperformed the lentiviral/VSV-G CD19-CAR vector, reaching up to 82.9% transduced NK cells (Figure 3C). The lentiviral/VSV-G vector reached similar transduction rates of CD19-CAR-NK cells compared to previous results (Figures 1D, 2A). In our study, MFIs of alpharetroviral/RD114-TR generated CD19-CAR-NK cells were significantly higher than those of lentiviral/VSV-G generated CD19-CAR-NK cells at MOI 5 and MOI 10 (Figure 3D), but did not differ at MOI 1. However, with higher MOI, the density of CAR-expression measured by MFI decreased following lentiviral/VSV-G CAR-transduction but increased following alpharetroviral/RD114-TR transduction (Figure 3D). Again, we analyzed the NK cell subpopulation distribution and their CD19-CAR expression. NK cell subpopulation distribution was neither influenced by lentiviral/VSV-G transduction nor by alpharetroviral/RD114-TR transduction (Supplementary Figure 8). CD19-CAR expression of CD16^+^ and CD16^−^ subpopulations did not significantly differ when compared within the viral vector system (Figure 3E) and matched with the CD19-CAR expression of the total NK cell population (Figure 3C). Overall, CD19-CAR expression of alpharetrovirally/RD114-TR transduced NK cell subpopulations outperformed those of lentiviral/VSV-G transduced NK cell subpopulations (Figures 3C–E).

### CD19-CAR-NK Cell Products Produce High Levels of Inflammatory Cytokines

**Figure 4:**
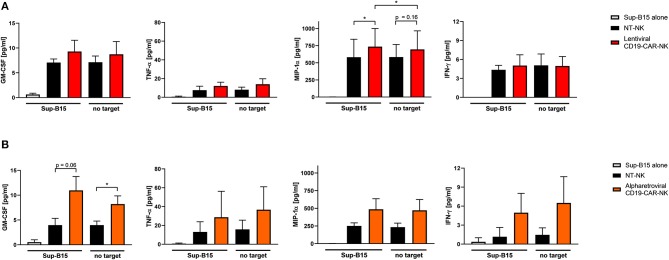
Cytokine secretion of CD19-CAR-NK cells. NT-NK cells, lentiviral/VSV-G CD19-CAR-NK cells and alpharetroviral/RD114-TR CD19-CAR-NK cells were cocultured with Sup-B15 cells at an E:T ratio of 1:1 for 4 h. As controls, NK cells and Sup-B15 cells were cultured alone. Levels of GM-CSF, TNF-α, MIP-1α, and IFN-γ within the supernatants were measured using a cytometric bead array. **(A)** Shows cytokine secretion of lentiviral/VSV-G CD19-CAR-NK cells in comparison to NT-NK cells from three donors *(n* = *3)*. Average transduction efficiency of used CD19-CAR-NK cells was 9.1% (range 7.3–11.6%). **(B)** Shows cytokine secretion of alpharetroviral/RD114-TR CD19-CAR-NK cells in comparison to NT-NK cells from three donors *(n* = *3)*. Average transduction efficiency of used CD19-CAR-NK cells was 15.8% (range 8.1–20.3%). Experiments in **(A)** were performed with different donors than experiments in **(B)**. Shown are mean values + SD. Statistical analysis was performed using two-tailed student's paired *t*-test. *p < 0.05. No significant differences were observed between analyzed groups without asterisks.

To further evaluate functional capacities of the CAR modified NK cells, cytokine production of GM-CSF, TNF-α, MIP-1α, and IFN-γ of lentivirally/VSV-G and alpharetrovirally/RD114-TR generated CD19-CAR-NK cells (both at MOI 5) was analyzed 3 days after transduction upon expansion in low dose IL-15 alone and in context of co-culturing with target-specific Sup-B15 ALL cells at an E:T ratio of 1:1 for 4 h. As controls, supernatant of Sup-B15 cells was analyzed. In general, CD19-CAR-NK cells tend to release more cytokines than NT-NK cells from the same donors regardless of target cell contact (Figure 4). This trend could be especially observed for CD19-CAR-NK cells transduced with lentiviral/VSV-G vectors (Figure 4A) for the release of MIP-1α and for CD19-CAR-NK cells transduced with alpharetroviral/RD114-TR vectors (Figure 4B) for the release of GM-CSF, TNF-α, MIP-1α, and IFN-γ. Of note, significant changes could only be observed for the release of MIP-1α of lentiviral/VSV-G CD19-CAR-NK cells upon contact with CAR specific target cells (Figure 4A) compared to NT-NK cells as well as compared to CD19-CAR-NK cells without target co-incubation. In the context of alpharetrovirally/RD114-TR transduced CD19-CAR-NK cells a slightly higher cytokine release of all analyzed cytokines could be shown, with significant changes only for GM-CSF (Figure 4B).

### Alpharetrovirally/RD114-TR Generated CD19-CAR-NK Cell Products Surpass Killing Efficiencies of Lentivirally/VSV-G Generated CD19-CAR-NK Cell Products

**Figure 5:**
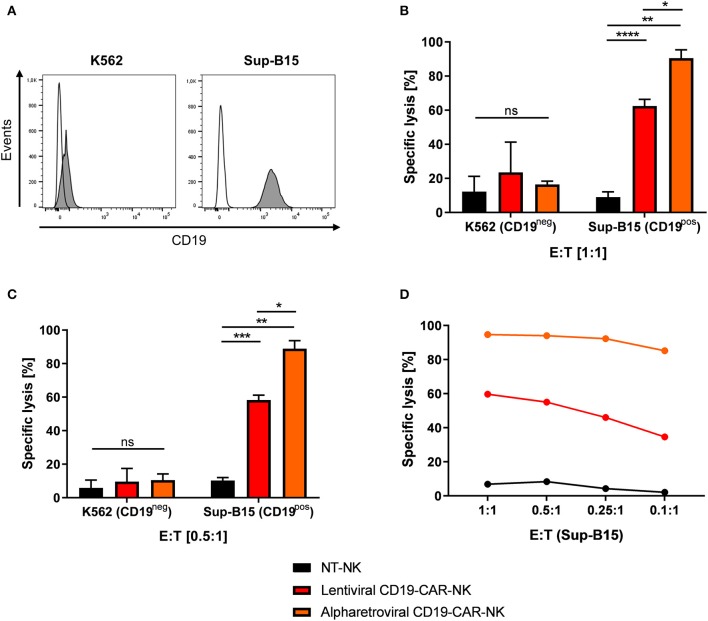
Comparison of cytotoxic capacity of CD19-CAR-NK cells generated by lentiviral/VSV-G or alpharetroviral/RD114-TR transduction. **(A)** CD19 expression on the surface of K562 and Sup-B15 leukemia cells was analyzed by flow cytometry with a CD19-specific antibody (filled areas). Unstained cells were used as controls (empty areas). **(B,C)** Cytotoxic activity of NT-NK cells, lentiviral/VSV-G CD19-CAR-NK cells and alpharetroviral/RD114-TR CD19-CAR-NK cells against K562 and Sup-B15 cells was determined in a flow cytometry-based cytotoxicity assay after a coincubation time of 4 h and with different E:T ratios. Average transduction efficiency of used lentiviral/VSV-G CD19-CAR-NK cells was 4.3% (range 2.9–6.9%), of used alpharetroviral/RD114-TR CD19-CAR-NK cells was 62.1% (range 56.1–69.6%). Shown are mean values + SD from co-incubations using NK cells of three different donors *(n* = *3)*. **(D)** Cytotoxic activity of NT-NK cells, lentiviral/VSV-G CD19-CAR-NK cells and alpharetroviral/RD114-TR CD19-CAR-NK cells against Sup-B15 at decreasing E:T ratios. Shown is a co-incubation using NK cells of one representative donor. Statistical analysis was performed using two-tailed student's paired *t*-test. ****p < 0.0001; ***p < 0.001; **p < 0.01; *p < 0.05, ns, not significant.

Next, the cytotoxic potential of lentivirally/VSV-G and alpharetrovirally/RD114-TR generated CD19-CAR-NK cells needed further assessment (scheme Figure 3A). For that, CD19-negative K562 erythroleukemia cells and CD19-expressing Sup-B15 ALL cells (Figure 5A) were stained with CFSE and used as targets in a 4 h flow cytometry-based cytotoxicity assay (for details see Materials and Methods). Lentivirally/VSV-G and alpharetrovirally/RD114-TR generated CD19-CAR-NK cells (both at MOI 5) and NT-NK cells were co-incubated with K562 or target-specific Sup-B15 for 4 h at E:T ratio of 1:1 (Figure 5B) or lower amount of effector cells (Figures 5C,D). In general, under assumption of normal distribution of NK cell function of healthy donors, CD19-CAR-NK cells showed a higher killing efficiency of CD19-expressing Sup-B15 cells than NT-NK cells (Figures 5B–D). At an E:T ratio of 1:1 the mean specific lysis of Sup-B15 was 90.5% for alpharetroviral/RD114-TR CD19-CAR-NK cells, 62.5% for lentiviral/VSV-G CD19-CAR-NK cells and 9.0% for NT-NK cells (Figure 5B). Interestingly, at an E:T ratio of 0.5:1 still comparable results were obtained (alpharetroviral/RD114-TR: 88.9%, lentiviral/VSV-G: 58.3%, NT-NK: 10.3%) (Figure 5C). As expected, lysis of CD19-negative K562 cells was on an equal level for all three NK cell preparations, without any significant difference between alpharetroviral/RD114-TR or lentiviral/VSV-G CD19-CAR-NK cells and NT-NK cells (Figures 5B,C). Mean transduction efficiencies of alpharetroviral/RD114-TR CD19-CAR-NK cells were 62.1% and 4.3% for lentiviral/VSV-G CD19-CAR-NK cells. In an additional assay, E:T ratios down to 0.1:1 were tested against Sup-B15 cells as targets. Even at low effector cell concentrations alpharetrovirally/RD114-TR generated CD19-CAR-NK cells remained highly cytotoxic and induced 85.2% lysed cells at an E:T ratio of 0.1:1 (Figure 5D). Contrary, lentiviral/VSV-G generated CD19-CAR-NK cells showed reduced cytotoxicity levels at lower effector cell numbers (Figure 5D). In this experiment, transduction efficiency of alpharetroviral/RD114-TR CD19-CAR-NK cells was 60.5% and 6.9% of lentiviral/VSV-G CD19-CAR-NK cells. Calculating the total number of CD19-CAR-NK cells in the NK cell products, comparable values were obtained for lentiviral/VSV-G CD19-CAR-NK cells at E:T ratio of 1:1 (total of 1725 CD19-CAR-NK cells) and alpharetroviral/RD114-TR CD19-CAR-NK cells at E:T ratio of 0.1:1 (total of 1512 CD19-CAR-NK cells). Notably, the comparison of specific lysis of these two conditions (lentiviral/VSV-G E:T 1:1 specific lysis of 59.7% Sup-B15 cells; alpharetroviral/RD114-TR E:T 0.1:1 specific lysis of 85.2% Sup-B15 cells) revealed that alpharetrovirally/RD114-TR transduced CD19-CAR-NK cells showed a higher cytotoxicity than lentiviral/VSV-G CD19-CAR-NK cells.

### Alpharetroviral and Lentiviral Vectors Perform Equally When Pseudotyped With RD114-TR

**Figure 6:**
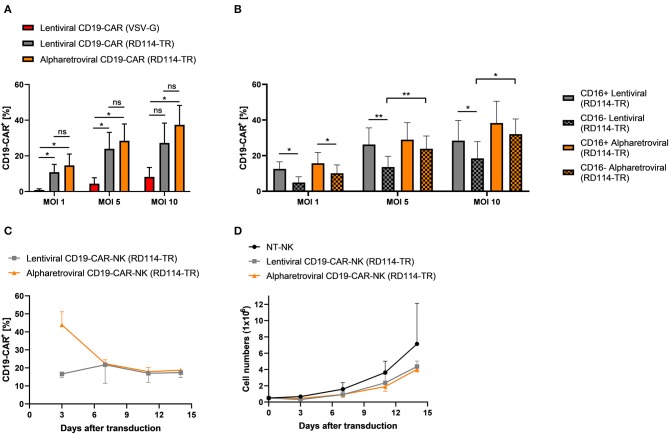
Transduction rates of lentivirally/RD114-TR modified NK cells and alpharetrovirally/RD114-TR modified NK cells do not differ. **(A)** Vectofusin-1 mediated transduction of NK cells from four donors *(n* = *4)* was performed with RD114-TR pseudotyped lentiviral CD19-CAR particles, RD114-TR pseudotyped alpharetroviral CD19-CAR particles or VSV-G pseudotyped lentiviral CD19-CAR particles at different MOIs. **(B)** CD19-CAR expression of CD16^+^ and CD16^−^ NK cell subpopulations. CD19-CAR expression of CD16^+^ and CD16^−^ NK cell subpopulations of lentiviral/RD114-TR and alpharetroviral/RD114-TR transduced cells depicted in **(A)** are shown *(n* = *4)*. **(C)** After transduction (day 0), CD19-CAR expression of lentiviral/RD114-TR and alpharetroviral/RD114-TR CD19-CAR-NK cells was determined by flow cytometry on four serial time points. Data from two donors (*n* = *2*) are shown. **(D)** Proliferation of NT-NK and CD19-CAR-NK cells was determined over a period of 14 days. Data from two donors (*n* = *2*) are shown. Shown are mean values + SD. Statistical analysis for **(A)** and **(B)** was performed using two-tailed student's paired *t*-test. **p < 0.01; *p < 0.05; ns, not significant.

Finally, we investigated if the observed high transduction efficiencies using alpharetroviral vectors pseudotyped with RD114-TR were related to the alpharetroviral vector system or to the RD114-TR envelope. Prior transduction on day 4 we observed upregulation of the sodium-dependent neutral amino acid transporter 2 (ASCT2, also known as SLC1A5) in NK cells pre-activated with low dose IL-15 (10 ng/ml) (Supplementary Figure 10). ASCT2 serves as receptor for the feline endogenous retrovirus RD114, of which the RD114-TR envelope was derived (44, 46). As ASCT2 was upregulated, we hypothesized that RD114-TR may be the reason for high transduction rates using alpharetroviral/RD114-TR vectors and concluded a lentiviral vector pseudotyped with RD114-TR might perform equally well. Therefore, NK cells from the same donor were transduced with the lentiviral CD19-CAR vector pseudotyped with RD114-TR (lentiviral/RD114-TR CD19-CAR). NK cells transduced with either the alpharetroviral/RD114-TR CD19-CAR vector or the lentiviral/VSV-G CD19-CAR vector served as controls. Across all MOIs lentiviral/RD114-TR transduction performed equally compared to alpharetroviral/RD114-TR transduction, as no significant differences were observed between both groups (Figure 6A). Additionally, the lentiviral/RD114-TR CD19-CAR vector could also outperform the lentiviral/VSV-G CD19-CAR vector at MOI 1 and MOI 5. Highest transduction efficiencies reached with the lentiviral/RD114-TR transduction was 40.6% (MOI 10). The alpharetroviral/RD114-TR CD19-CAR vector performed similar and the highest efficiency it reached was 47.7% (MOI 10). Once more, we analyzed the NK cell subpopulation distribution and their CD19-CAR expression. NK cell subpopulation distribution was neither influenced by lentiviral transduction nor by alpharetroviral transduction (Supplementary Figure 9, data for lentiviral/VSV-G not shown). Interestingly, in the NK cell population transduced with the lentiviral/RD114-TR CD19-CAR vector, CD16^+^ cells showed a higher CD19-CAR expression (Figure 6B). For NK cells transduced with the alpharetroviral/RD114-TR CD19-CAR vector this could only be observed at MOI 1, as at higher MOIs comparable results to Figure 3E were achieved and CD19-CAR expression of CD16^+^ and CD16^−^ subpopulations did not significantly differ. Overall, CD19-CAR expression of alpharetrovirally/RD114-TR transduced NK cell populations were similar to those of lentivirally/RD114-TR transduced NK cell populations. Exceptions were observed for MOI 5 and MOI 10, as the alpharetroviral CD16^−^ subpopulation expressed more CD19-CAR than the lentiviral CD16^−^ subpopulation.

Next, we investigated transgene expression and expansion of generated CD19-CAR-NK cells over time. Therefore CD19-CAR-NK cells were generated using the lentiviral/RD114-TR CD19-CAR vector and the alpharetroviral/RD114-TR vector at MOIs 3–5, kept in culture with 10 ng/ml IL-15 and were analyzed by flow cytometry and counted every 3–4 days. First, we observed a decrease of the CD19-CAR expression for alpharetrovirally/RD114-TR generated CD19-CAR-NK cells (Figure 6C). But later on, CD19-CAR expression remained on a stable level for these cells. Lentivirally/RD114-TR generated CD19-CAR-NK cells expressed the CD19-CAR on an evenly level throughout the 14 days observation time (Figure 6C). Interestingly one donor could even increase the CD19-CAR expression level (from 15.3% on day 3 to 19.3% on day 14). Expansion of CD19-CAR-NK cells did not differ to NT-NK cells from the same donors, even though a slight better expansion could be observed for NT-NK cells (Figure 6D).

### Functional and Cytotoxic Capacities of Lentivirally/RD114-TR Generated CD19-CAR-NK Cells Are Comparable to Alpharetrovirally/RD114-TR Generated CD19-CAR-NK Cells

**Figure 7:**
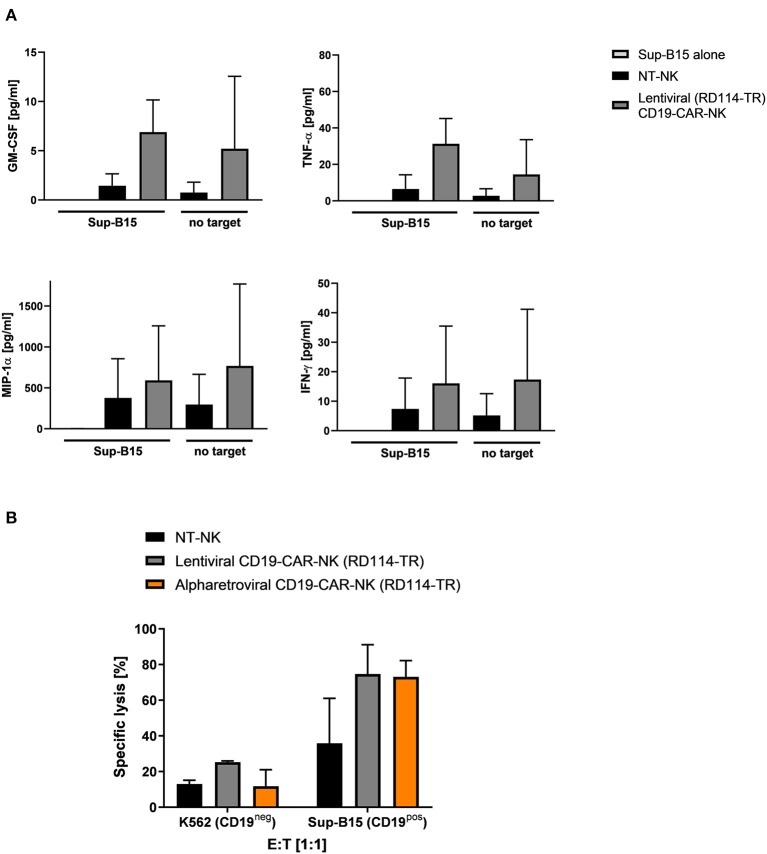
Cytokine secretion and cytotoxic capacity of CD19-CAR-NK cells generated by RD114-TR pseudotyped lentiviral CD19-CAR particles. **(A)** NT-NK cells and lentiviral/RD114-TR CD19-CAR-NK cells were cocultured with Sup-B15 cells at an E:T ratio of 1:1 for 4 h. As controls, NK cells and Sup-B15 cells were cultured alone. Levels of GM-CSF, TNF-α, MIP-1α, and IFN-γ within the supernatants were measured using a cytometric bead array. Average transduction efficiency of used lentiviral/RD114-TR CD19-CAR-NK cells was 13.1% (range 11.6–14.5%). Cytokine secretion from two donors *(n* = *2)* are shown. **(B)** Cytotoxic activity of NT-NK cells, lentiviral/RD114-TR CD19-CAR-NK cells and alpharetroviral/RD114-TR CD19-CAR-NK cells against K562 and Sup-B15 cells was determined in a flow cytometry-based cytotoxicity assay after a coincubation time of 4 h at an E:T: ratio of 1:1. Average transduction efficiency of used lentiviral/RD114-TR CD19-CAR-NK cells was 13.1% (range 11.6–14.5%), of used alpharetroviral/RD114-TR CD19-CAR-NK cells was 23.4% (range 22.6–23.9%). Shown are co-incubations using NK cells of two different donors *(n* = *2)*. Shown are mean values + SD.

To further evaluate functionality and cytotoxicity of lentivirally/RD114-TR generated CD19-CAR-NK cells (MOI 3–5), cytokine production and cytotoxicity of these cells was evaluated. Cytokine production of GM-CSF, TNF-α, MIP-1α, and IFN-γ of lentiviral/RD114-TR CD19-CAR-NK cells was analyzed 3 days after transduction upon expansion in low dose IL-15 alone and in context of co-culturing with target-specific Sup-B15 ALL cells at an E:T ratio of 1:1 for 4 h. As controls, supernatant of Sup-B15 cells was analyzed. In general, we observed similar results as shown in Figure 4: lentivirally/RD114-TR generated CD19-CAR-NK cells tend to release more cytokines than NT-NK cells from the same donors regardless of target cell contact (Figure 7A). To analyze cytotoxic capacities of lentiviral/RD114-TR generated CD19-CAR-NK cells (MOI 3–5), we performed cytotoxicity assays against CD19-negative K562 erythroleukemia cells and CD19-expressing Sup-B15 ALL cells as target cells. NT-NK cells and alpharetrovirally/RD114-TR generated CD19-CAR-NK cells (MOI 3–5) of the same donor served as controls and coincubations were performed at an E:T ratio of 1:1. Once more, CD19-CAR-NK cells showed a higher killing efficiency of CD19-expressing Sub-B15 cells than NT-NK cells (Figure 7B). Mean specific lysis of Sup-B15 cells was 74.7% for lentiviral/RD114-TR CD19-CAR-NK cells, 73.1% for alpharetroviral/RD114-TR CD19-CAR-NK cells, and 35.9% for NT-NK cells. Lysis of K562 cells was on an equal level for all three NK cell preparations. Mean transduction efficiencies of lentiviral/RD114-TR CD19-CAR-NK cells were 13.1% and 23.3% for alpharetroviral/RD114-TR CD19-CAR-NK cells. The lower transduction efficiencies might explain the lower observed cytotoxic capacities compared to Figure 5B. Overall, in this setting lentivirally/RD114-TR generated CD19-CAR-NK cells performed equally compared to alpharetrovirally/RD114-TR generated CD19-CAR-NK cells.

## Discussion

NK cells modified to express a CD19-specific CAR represent an allogenic alternative to CD19-CAR-T cells for the therapy of relapsed and refractory B-ALL. However, genetic modification of PB-derived NK cells to express a CAR remains difficult. Here, we investigated different ways for genetic engineering of PB-derived NK cells to express a CD19-specific CAR by retroviral vectors. Highest transduction efficiencies were achieved with retroviral CD19-CAR vectors pseudotyped with RD114-TR envelope and combined with Vectofusin-1 as transduction enhancer, generating functional and highly cytotoxic CD19-CAR-NK cells.

Vectofusin-1, also known as LAH4-A4, is a cationic peptide derived from the LAH4 peptide family (47). Initially, LAH4 peptides were used as DNA transfection agents (48), but recent works showed the ability of Vectofusin-1 to promote transduction of human CD34^+^ hematopoietic stem and progenitor cells (hCD34^+^ HSPCs) and human T cells using a broad range of lentiviral and gammaretroviral pseudotypes, including RD114-TR and at least for hCD34^+^ HSPCs also VSV-G (47, 49, 50). Thereby, Vectofusin-1 was at least as effective as Retronectin in enhancing transduction, and in some cases more effective (47, 50). The two substances act differently enhancing gene delivery into target cells: Retronectin on the one hand is a fibronectin fragment that facilitates colocalization of viruses and target cells by binding viral particles and target cells with different domains (51). On the other hand the exact mechanism of Vectofusin-1 is not completely explored, but it was shown that Vectofusin-1 forms α-helical coiled-coil fibrils that sediment lentiviral particles and therefore increase the local virus concentration along the surface of target cells (52). Main advantage of using Vectofusin-1 instead of Retronectin is its simple usage, because Vectofusin-1 is added directly into the transduction mixture. In comparison, Retronectin needs a pre-coating step, which is more difficult to standardize and extends the transduction process. Comparing both substances in their ability to enhance the transduction of human NK cells, we could show that Vectofusin-1 is as effective as Retronectin in transduction of NK cells using VSV-G pseudotyped lentiviral vectors. Interestingly, in the context of alpharetroviral transduction using a RD114-TR pseudotyped EGFP vector, Vectofusin-1 promoted transduction of NK cells better than Retronectin. Overall, we demonstrated for the transduction of PB-derived NK cells that Vectofusin-1 serves as a promising potential alternative to the frequently used Retronectin.

The second component of the most successful protocol in this study was pseudotyping alpharetroviral and lentiviral vectors with RD114-TR. The alpharetroviral vector system is based on self-inactivating (SIN) alpharetroviral vectors with a split-packaging design (40). These alpharetroviral vectors were successfully used to genetically modify NK cells (45, 53, 54) and outperformed lentiviral and gammaretroviral transduction efficiencies of PB-derived NK cells using EGFP encoding vectors (45). Furthermore, due to its more random integration pattern with lower frequency of integrations in or close to gene coding regions, alpharetroviral vectors can be considered to be safer as insertional oncogenesis is less likely to occur (55, 56). Initially, we observed a superiority of alpharetroviral/RD114-TR transduction of NK cells compared to lentiviral/VSV-G transduction, using second-generation CD19-CAR encoding vectors. To confirm this, we compared alpharetroviral/RD114-TR transduction of NK cells to lentiviral/RD114-TR transduction. Thereby, no superiority of one system could be observed as both viral vector systems performed equally well. Additionally, lentiviral/RD114-TR transduction of NK cells also outperformed lentiviral/VSV-G transduction. Interestingly, these data suggest that the key for a high transduction rate was the pseudotyping of retroviral vectors with RD114-TR. Our findings are consistent with a recent publication by Bari et al., which showed that PB-derived NK cells were poorly transduced by lentiviral/VSV-G based transduction (57). But using a modified baboon envelope glycoprotein (BaEV) (58) to pseudotype lentiviral CD19-CAR particles, an average transduction efficiency of 70% could be reached and ASCT-2 has been proposed as the entry receptor for BaEV (59). Interestingly, ASCT-2 is also the receptor for feline endogenous retrovirus RD114 based envelopes (46). Indeed, we observed high expression of ASCT-2 in low dose IL-15 activated NK cells on the day of transduction. This may explain the success of the RD114-TR envelope and demonstrates how important the knowledge of receptor expression of NK cells is for an effective transduction.

Both CD19-CAR-NK cell preparations generated by RD114-TR pseudotyped retroviral vectors proliferated similar and no significant differences compared to NT-NK cells could be observed. Furthermore, 7–11 days after the transduction procedure, transduced NK cells showed still a stable expression of the CD19-CAR. Interestingly, CD19-CAR expression of alpharetrovirally transduced NK cells declined in the first 7–11 days, but afterwards a certain plateau was reached. Similar CD19-CAR expression could be observed for lentiviral/RD114-TR and alpharetroviral/RD114-TR transduced NK cells at the end of the observation period. Overall, these results indicate that both protocols are suitable to generate a sufficient number of CD19-CAR-NK cells for clinical applications, as the CD19-CAR is stable expressed and transduced cells proliferate as good as NT-NK cells.

Regarding functionality and killing activity, we addressed cytokine production and cytotoxic capacity of CD19-CAR-NK cells transduced with different methods. Independently of a direct contact with the CD19-expressing target cells, CD19-CAR-NK cells tend to release more cytokines than NT-NK cells. This tendency could be observed as a slight increase in production of MIP-1α in lentivirally/VSV-G transduced CD19-CAR-NK cells, and GM-CSF, TNF-α, MIP-1α, and IFN-γ in alpharetrovirally/RD114-TR and lentivirally/RD114-TR transduced CD19-CAR-NK cells. However, significant changes could only be observed for the release of MIP-1α of lentiviral/VSV-G CD19-CAR-NK cells and for GM-CSF in alpharetrovirally/RD114-TR transduced CD19-CAR-NK cells compared to NT-NK cells. These observations could be explained by possible influence of genetic engineering using retroviral vectors, as these changes occurred in transduced cells independent of direct contact with target-specific cells.

Concerning the direct cytotoxic potential against CD19-expressing Sup-B15 in comparison to the non-target specific cytotoxicity against K562 leukemia cells, alpharetrovirally/RD114-TR generated CD19-CAR-NK cells displayed higher cell killing activity than lentivirally/VSV-G generated CD19-CAR-NK cells. This is most likely in part due to the fact that more NK cells were transduced using the alpharetroviral vector, as lentivirally/RD114-TR generated CD19-CAR-NK cells with a similar transduction efficiency as alpharetrovirally/RD114-TR generated CD19-CAR-NK cells showed equally good cytotoxic activities against Sup-B15 cells. But if similar numbers of CD19-CAR-NK cells are applied independent of the used vector, CD19-CAR-NK cells generated with the alpharetroviral vector show a better killing performance than those generated by lentiviral/VSV-G transduction. Of note, lentivirally/VSV-G transduced CD19-CAR-NK cells with a significantly lower mean transduction efficiency showed already around 60% killing of Sup-B15 cells. This might point either to an impressively high lytic capacity of each single lentivirally transduced NK cell or to a high and so far underestimated antigen-independent killing mechanism of NT-NK bystander cells. Overall our results demonstrate that, independent of the used retroviral vector for the genetic modification of NK cells, a relative low number of CD19-CAR-NK cells can kill a sufficient amount of target-specific tumor cells.

In general, the remarkably high killing capacity could be explained by serial killing activity of NK cells, as one NK cell is able to hit multiple targets (60, 61). It has been shown that serial killing of tumor cells by cytokine activated CD16^+^ NK cells is increased in the presence of rituximab (62). Therefore, a combination of CD19-CAR-NK cells possessing ADCC with the anti-CD20 monoclonal antibody Rituximab may provide a treatment option for aggressive relapsed CD19^+^ and CD20^+^ B-ALL. Pre-clinical data have shown synergistic tumor suppressing action using CD19-CAR-T cells with rituximab on mice inoculated with B cell non-Hodgkin lymphoma (B-NHL) (63). Interestingly, the authors considered presence of contaminating NK cells in their setting as the cause for the synergistic effect, supporting our approach to utilize CAR-NK cells for immunotherapy of malignant diseases.

As CD16 expression is crucial for exerting ADCC, we investigated CD16 expression of transduced NK cells and their transducability. Consistent with previous other findings (own unpublished data), we observed a decline of the CD16^+^ subpopulation in NK cells with advancing time of cell cultivation. However, our data here demonstrated that the CD16 proportion of transduced PB-derived NK cells is not influenced by the genetic engineering. Concerning lentiviral/VSV-G and alpharetroviral/RD114-TR transduced NK cells, CD19-CAR expression of CD16^+^ and CD16^−^ subpopulations did not differ, indicating that there is no favorable NK cell subpopulation for genetic engineering by retroviral vectors. Interestingly, the CD16^+^ subpopulation was more susceptible to the lentiviral/RD114-TR CD19-CAR vector as their transduction rate was significantly higher than its CD16^−^ counterpart.

In sum, our protocol to generate IL-15 activated CD19-CAR-NK cells led to increased cytotoxic activity against CD19-expressing target cells when compared to NT-NK cells. Alpharetrovirally/RD114-TR and lentivirally/RD114-TR transduced NK cells were highly cytotoxic, with alpharetrovirally/RD114-TR transduced NK cells achieving 90% specific killing activity. Moreover, our findings are the first ones that indicate the use of Vectofusin-1 in combination with alpharetroviral/RD114-TR vectors as a successful strategy to genetically modify PB-derived NK cells to achieve highly cytotoxic CD19-CAR-NK cells at high yields. Furthermore, we could show that lentiviral/RD114-TR vectors in combination with Vectofusin-1 act as a suitable alternative. With these newly developed NK cell expansion and transduction protocols, gene modified NK cell products can be prepared off-the-shelf that might open new avenues for cancer immunotherapy.

## Data Availability Statement

All datasets generated for this study are available on request to the corresponding author.

## Ethics Statement

The studies involving human participants were reviewed and approved by the Ethics Committee of the Goethe University Frankfurt, Germany (approval no. 329/10). The patients/participants provided their written informed consent to participate in this study.

## Author Contributions

EU designed the project. SM, TB, VG, FK, and ES performed experiments and analyzed data. AS and WW provided plasmids. JH, AS, UK, WW, and UM provided expert input. SM, TB, JH, AS, UK, WW, UM and EU discussed data. SM, UM, and EU wrote the manuscript with support from all other co-authors. All authors agree to be accountable for the content of the work.

### Conflict of Interest

WW (CD19-CAR sequence in the alpharetroviral and lentiviral CD19-CAR vector) is named as an inventor in patents and patent applications on CAR technology owned by Georg-Speyer-Haus. AS is named as an inventor on a patent describing alpharetroviral SIN vectors (alpharetroviral backbone sequence and used alpharetroviral helper plasmid). The remaining authors declare that the research was conducted in the absence of any commercial or financial relationships that could be construed as a potential conflict of interest. The reviewer EV declared a past co-authorship with several of the authors, WW, UM, to the handling Editor.

## Abbreviations

ADCC: antibody-dependent cell-mediated cytotoxicity
ASCT-2: acronym for Alanine, Serine, Cysteine Transporter 2. A sodium-dependent neutral amino acid transporter
BaEV: modified baboon envelope glycoprotein
B-ALL: B-cell lymphoblastic acute leukemia
BSA: bovine serum albumin
CAR: chimeric antigen receptor
CBA: cytometric bead array
CD: cluster of differentiation
CFSE: carboxyfluorescein succinimidyl ester
DAPI: 4′,6-diamidino-2-phenylindole
DMEM: Dulbecco's Modified Eagle Medium
E:T: effector to target ratio
EGFP: enhanced green fluorescence protein
FBS: fetal bovine serum
GM-CSF: granulocyte-macrophage colony-stimulating factor
GvHD: graft vs. host disease
HBSS/HEPES: Hank's balanced salt solution containing 2,5% of 1M HEPES, 4-(2-hydroxyethyl)-1-piperazineethanesulfonic acid
HEK: human embryonic kidney
HLA: human leukocytes antigen
IFN-γ: interferon gamma
IL-15: interleukin 15
LV: lentiviral
MFI: mean fluorescence intensity
MIP-1α: macrophage inflammatory protein 1-alpha
MOI: multiplicity of infection, NK cell, natural killer cell
ns: not significant
NT-NK cell: non-transduced natural killer cell
PB: peripheral blood
PBMC: peripheral blood mononuclear cell
PBS: phosphate-buffered saline
Pen/Strep: Penicillin and Streptomycin
RD114-TR: modified version of the feline endogenous retrovirus (RD-114 virus) envelope
RPMI: Roswell Park Memorial Institute
scFv: single-chain variable fragment
SD: standard deviation
SFFV: Spleen Focus-Forming Virus
SIN: Self-Inactivating
SP: signal peptide
TNF-α: tumor necrosis factor alpha
VSV-G: vesicular stomatitis virus G glycoprotein
α: alpharetroviral.
