# Supplementary material for: High Cytotoxic Efficiency of Lentivirally and Alpharetrovirally Engineered CD19-Specific Chimeric Antigen Receptor Natural Killer Cells Against Acute Lymphoblastic Leukemia
Source: Front Immunol. 2020 Jan 24;10:3123. doi: 10.3389/fimmu.2019.03123 (PMC7025537; doi:10.3389/fimmu.2019.03123)
Supplement: Supplementary file 1 [file Data_Sheet_1.pdf]

Supplementary Material

High cytotoxic efficiency of lentivirally and alpharetrovirally engineered CD19-specific chimeric antigen receptor natural killer cells against acute lymphoblastic leukemia

Stephan Müller, Tobias Bexte, Veronika Gebel, Franziska Kalensee, Eva Stolzenberg, Jessica Hartmann, Ulrike Koehl, Axel Schambach, Winfried S. Wels, Ute Modlich, Evelyn Ullrich

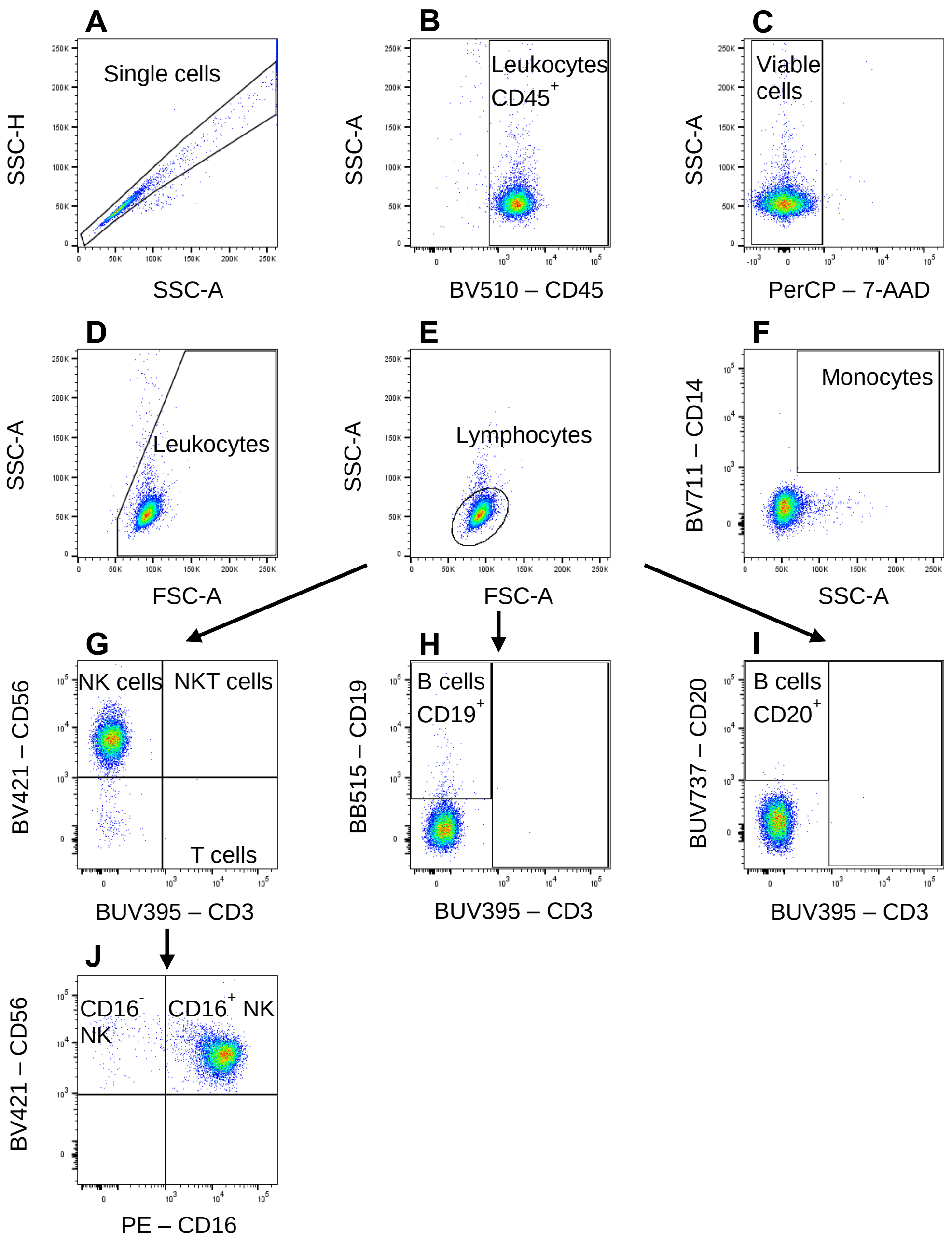

Supplementary Figure 1: Flow cytometric gating strategy to demonstrate purity of NK cells and distinct NK cell subpopulations.

Shown are NK cells after isolation from PBMCs (NK cell enrichment). The first SSC-A/SSC-H plot separates single cells from duplicates **(A)**. The next plot identifies all $CD45^+$ cells (Leukocytes) **(B)**. The third gate focusses all viable cells by excluding all 7-AAD^+ events **(C)**. After that all viable leukocytes are identified based on their size and granularity within the FSC-A/SSC-A plot **(D)**. From the gate in **(D)** all monocytes are identified based on their granularity and CD14 expression **(F)**, as well as lymphocytes based on their size and granularity **(E)**. Finally, from the lymphocytes **(E)** NK cells ($CD56^+$ $CD3^-$), NKT cells ($CD56^+$ $CD3^+$), T-cells ($CD56^-$ $CD3^+$) and B-cells ($CD19^+$ $CD3^-$ or $CD20^+$ $CD3^-$) are discriminated **(G, H, I)**. At last NK cells are divided regarding their CD16 expression **(J)**.

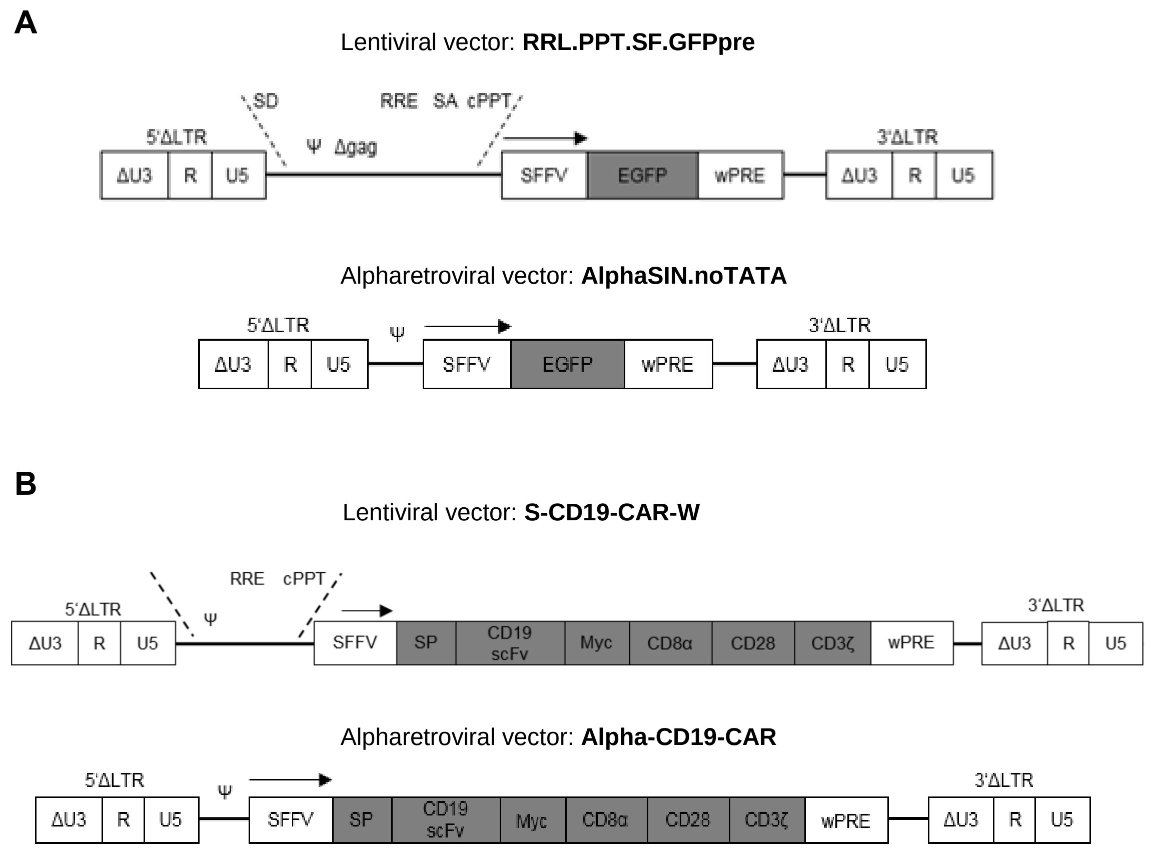

Supplementary Figure 2: Schematic illustration of retroviral SIN vectors.

(A) EGFP vectors: The EGFP transgene depticted in dark grey under control of the Spleen Focus-Forming Virus (SFFV) promotor. It was flanked by the long terminal-repeat (LTR) (ΔU3, R, U5) sequences. Additionally, the woodchuck hepatitis post-transcriptional regulatory element (wPRE), the splice donor and acceptor site (SD and SA), the packaging signal (Ψ), a remaining gag-coding sequence (Δgag), the rev-responsive element (RRE) and the central poly-purine tract (cPPT) are shown. Modified after *Suerth et al.*[1]

(B) CD19-CAR vectors: The anti-CD19-CAR transgene depticted in dark grey consists of an immunoglobulin heavy-chain signal peptide (SP), the anti-CD19 scFv domain of a mouse monoclonal antibody (FMC63), a myc-tag (Myc), a CD8α hinge region, the CD28 transmembrane domain, and a composite CD28-CD3ζ signaling domain, expressed under the control of the SFFV promotor. It was flanked by the long terminal-repeat (LTR) (ΔU3, R, U5) sequences. Remaining elements are indicated in **(A)**. Lentiviral vector modified after *Oelsner et al.*[2] Integrated proviral forms of the vectors are shown.

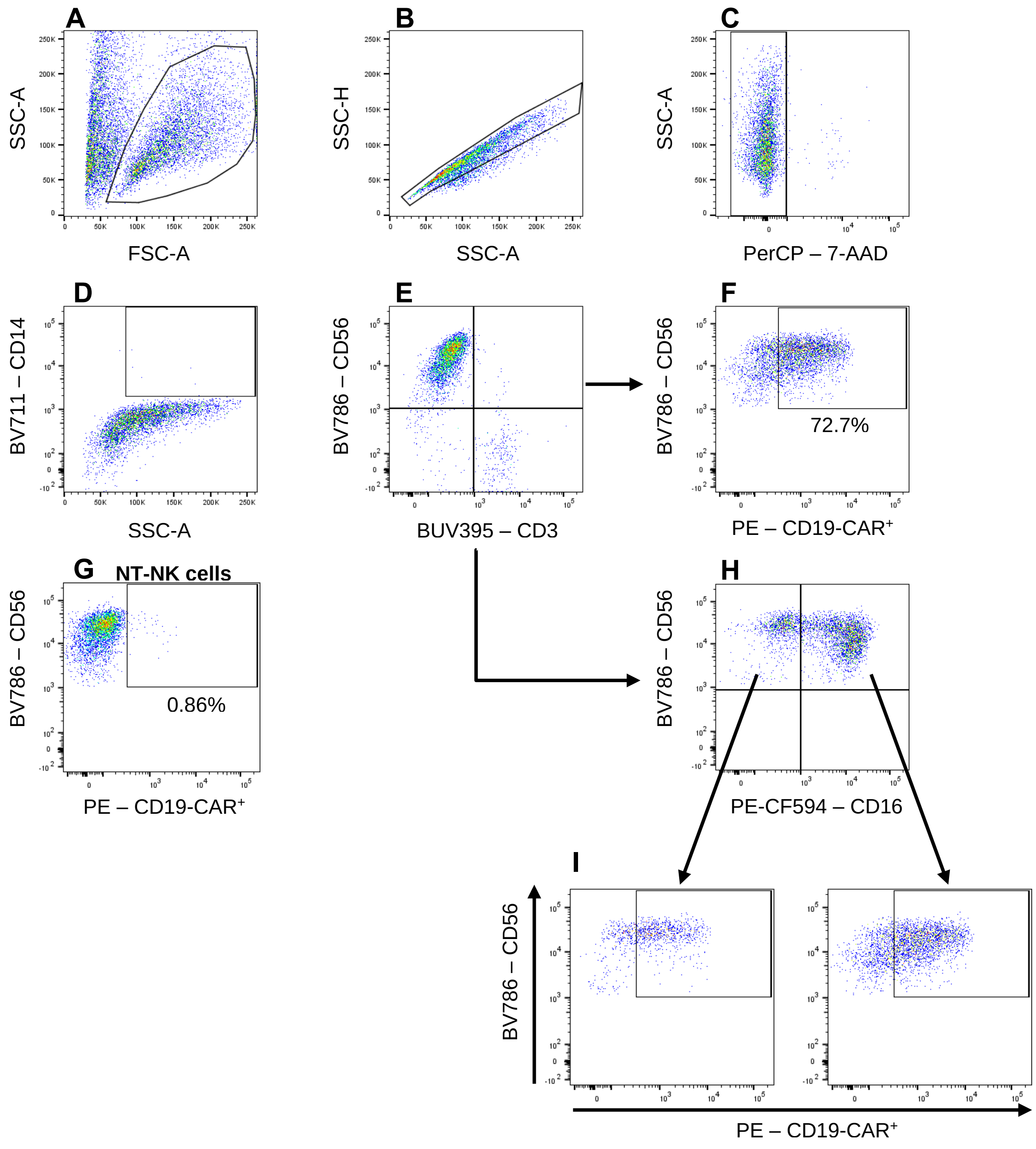

Supplementary Figure 3: Flow cytometric analysis to estimate transduction efficiency of transduced NK cells.

Shown is a transduction with RD114-TR pseudotyped alpharetroviral CD19-CAR particles at a multiplicity of infection (MOI) of 10 **(A – F, H – I)**. Non-transduced (NT) NK cells of the same donor were used as a reference **(G)**. The first FSC-A/SSC-A plot identified all leukocytes and excluded debris **(A)**. After that duplicates were excluded **(B)** and all viable cells were focused on **(C)**. From the gate in **(C)** monocytes were identified based on granularity and CD14 expression **(D)**, as well as NK cells ($CD56^+$ $CD3^-$), NKT cells ($CD56^+$ $CD3^+$) and T cells ($CD56^-$ $CD3^+$) **(E)**. From the NK cell population CD19-CAR positive events were measured via a myc-tag fluorescent antibody (PE) **(F)**. As a reference where to gate, stained NT-NK cells from the same donor were used **(G)**. Positivity in **(G)** was subtracted from positivity in **(F)** to calculate the total amount of $CD19\text{-}CAR^+$ NK cells. At last, NK cells were divided regarding their CD16 expression and CD19-CAR expression of these subpopulations was determined **(H and I)**.

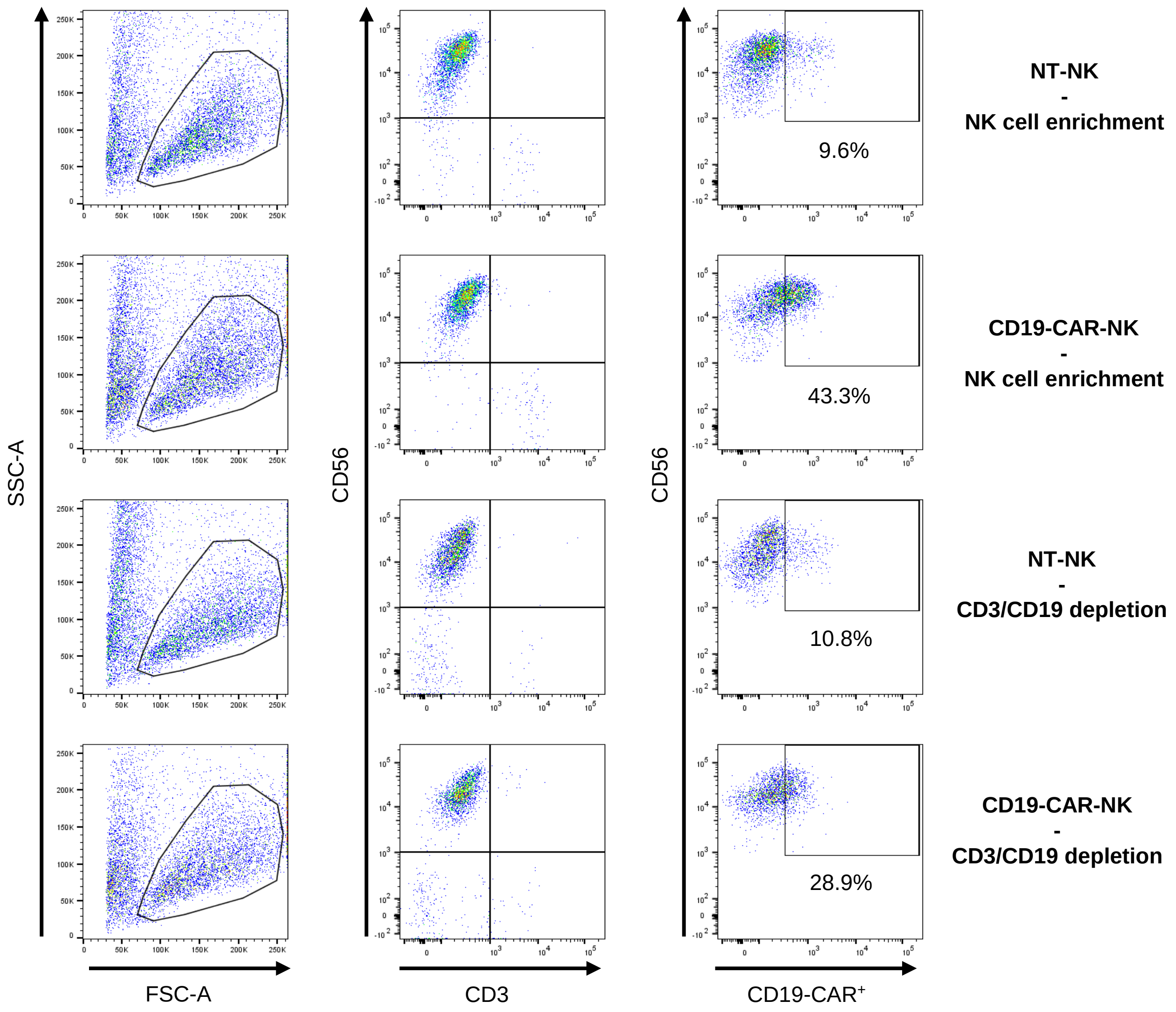

Supplementary Figure 4: Representative dot plots of NK cells isolated by NK cell enrichment or CD3/CD19 depletion transduced with VSV-G pseudotyped lentiviral CD19-CAR particles as shown in Figure 2A.

Shortened gating strategy to estimate the transduction efficiency of NK cells isolated by NK cell enrichment (first and second row) or CD3/CD19 depletion (third and fourth row) transduced with VSV-G pseudotyped lentiviral CD19-CAR particles at MOI 10. NK cells were identified as $CD56^{+}CD3^{-}$ leukocytes (first and second column). From those $CD19\text{-}CAR^{+}$ NK cells were estimated (third column). In the first and second row representative data of NK cells isolated by NK cell enrichment are depicted that were transduced with Vectofusin-1 at MOI 10 versus non-transduced (NT) NK cells from NK cell preparations of the same donor. In the third and fourth row data from NK cells isolated by CD3/CD19 depletion transduced with Vectofusin-1 at MOI 10 versus NT-NK cells are shown. Percentage of false positive CD19-CAR events in NT-NK cells was subtracted from the percentages measured in the belonging transduced NK cells. Shown are the dot plots of one donor.

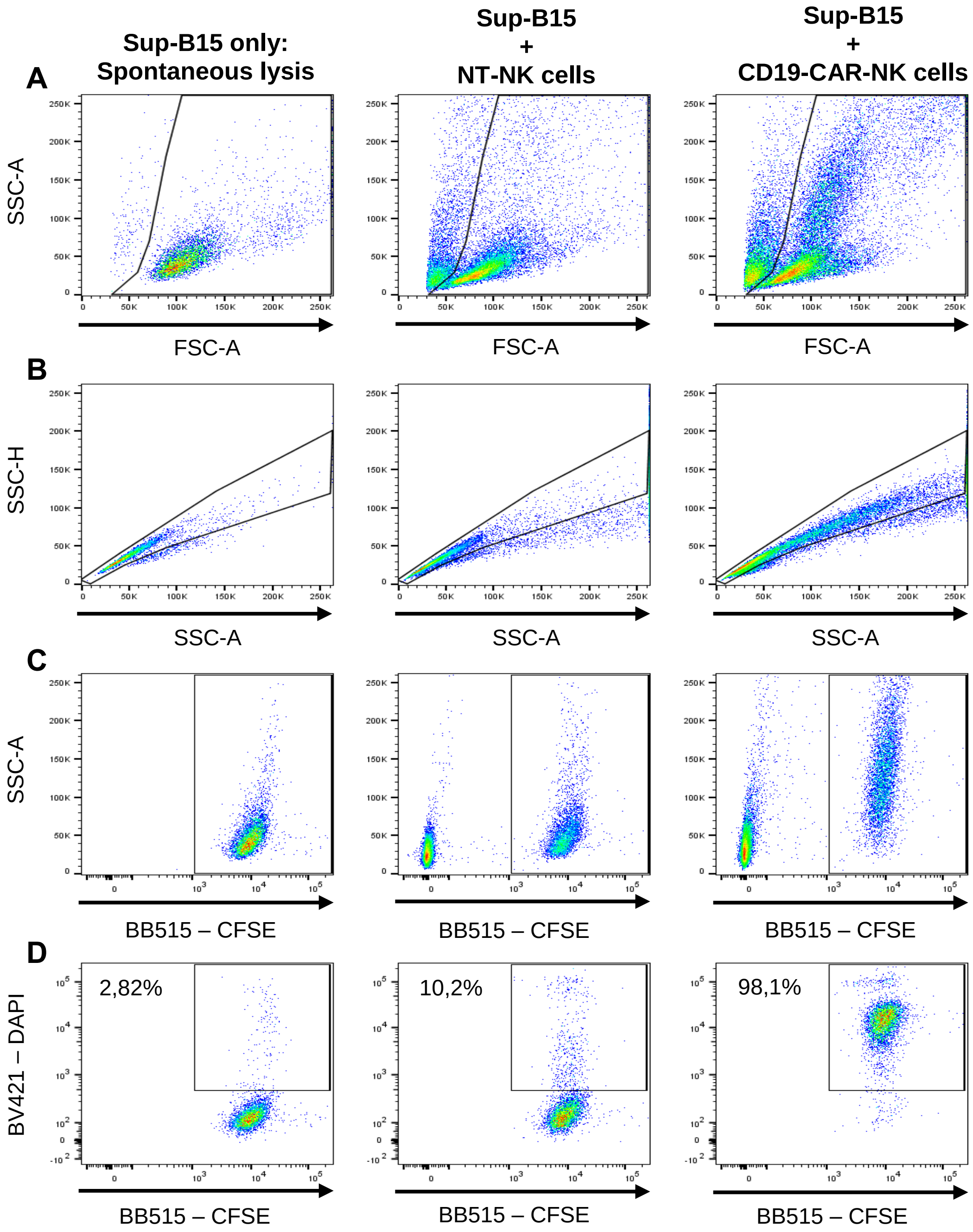

Supplementary Figure 5: Flow cytometric cytotoxicity assay of Sup-B15.

CD19-expressing Sup-B15 cells (ALL) were stained with CFSE and cocultured with NT-NK cells and CD19-CAR-NK cells from the same donor at different effector to target (E:T) ratios for four hours (second and third column). After coincubation cells were resuspended in DAPI solution and analyzed by flow cytometry. Samples only containing target cells were used as spontaneous cell lysis controls (first column). First all events were identified except small debris and fragments **(A)**. After that single cells were gated **(B)** and only CFSE$^+$ cells were focused on **(C)**. At last dead CFSE$^+$ cells were identified as CFSE$^+$ and DAPI$^+$ **(D)**. Specific lysis of Sup-B15 cells mediated by NK cells was calculated by subtracting spontaneous lysis values from measured target cell lysis in **(D)**. Shown is a coincubation of NK cells with Sup-B15 cells at E:T ratio of 1:1.

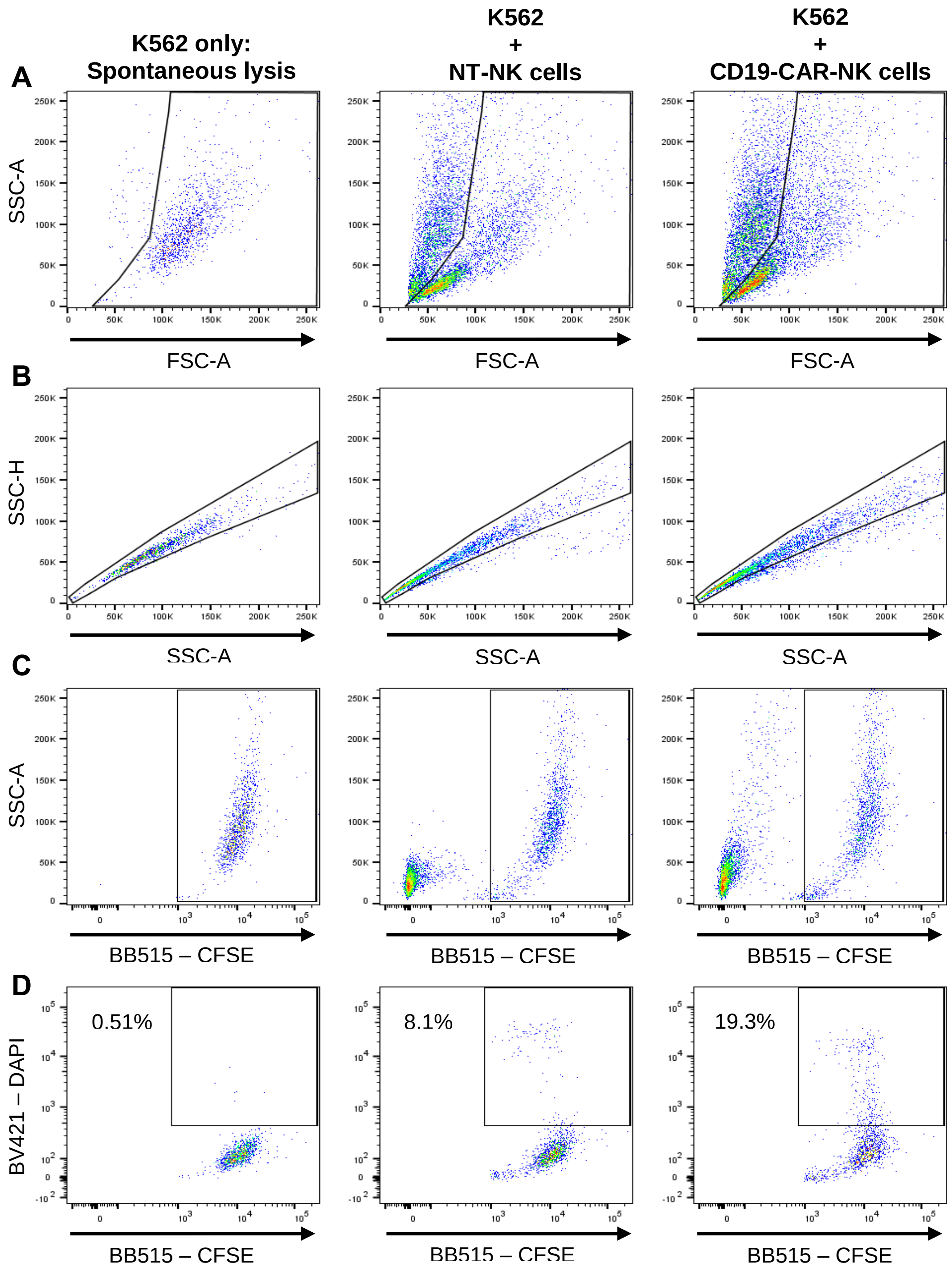

Supplementary Figure 6: Flow cytometric cytotoxicity assay of K562.

CD19-negative K562 cells (CML) were stained with CFSE and cocultured with NT-NK cells and CD19-CAR-NK cells from the same donor at different effector to target (E:T) ratios for four hours (second and third column). After coincubation cells were resuspended in DAPI solution and analyzed by flow cytometry. Samples only containing target cells were used as spontaneous cell lysis controls (first column). First all events were identified except small debris and fragments **(A)**. After that single cells were gated **(B)** and only CFSE$^+$ cells were focused on **(C)**. At last dead CFSE$^+$ cells were identified as CFSE$^+$ and DAPI$^+$ **(D)**. Specific lysis of K562 cells mediated by NK cells was calculated by subtracting spontaneous lysis values from measured target cell lysis in **(D)**. Shown is a coincubation of NK cells with K562 cells at E:T ratio of 1:1.

A

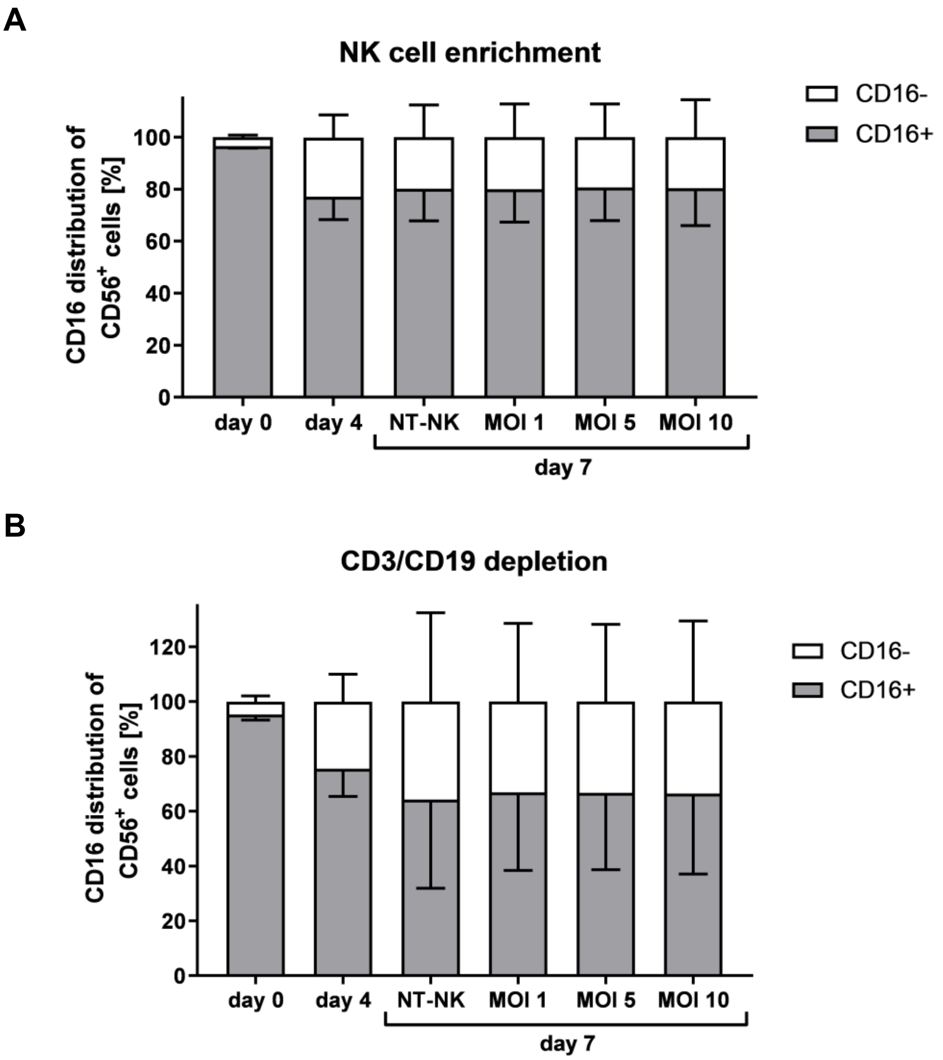

Supplementary Figure 7: CD16 expression of NK cells transduced with the lentiviral CD19-CAR using Vectofusin-1 as shown in Figure 2A.

CD16 expression of NK cells ($CD56^{+}CD3^{-}$) was determined on day of isolation (day 0), prior transduction (day 4) and on day 7, as transduction efficiency was analyzed. **(A)** shows CD16 expression of NK cells isolated by NK cell enrichment and transduced on day 4 using Vectofusin-1 and VSV-G pseudotyped lentiviral CD19-CAR particles. **(B)** shows CD16 expression of NK cells isolated by CD3/CD19 depletion and transduced on day 4 using Vectofusin-1 and VSV-G pseudotyped lentiviral CD19-CAR particles. On day 7 NT-NK cells were analyzed as controls. NK cells in **(A)** and **(B)** were derived from four different donors *(n = 4)*. Shown are mean values ± SD.

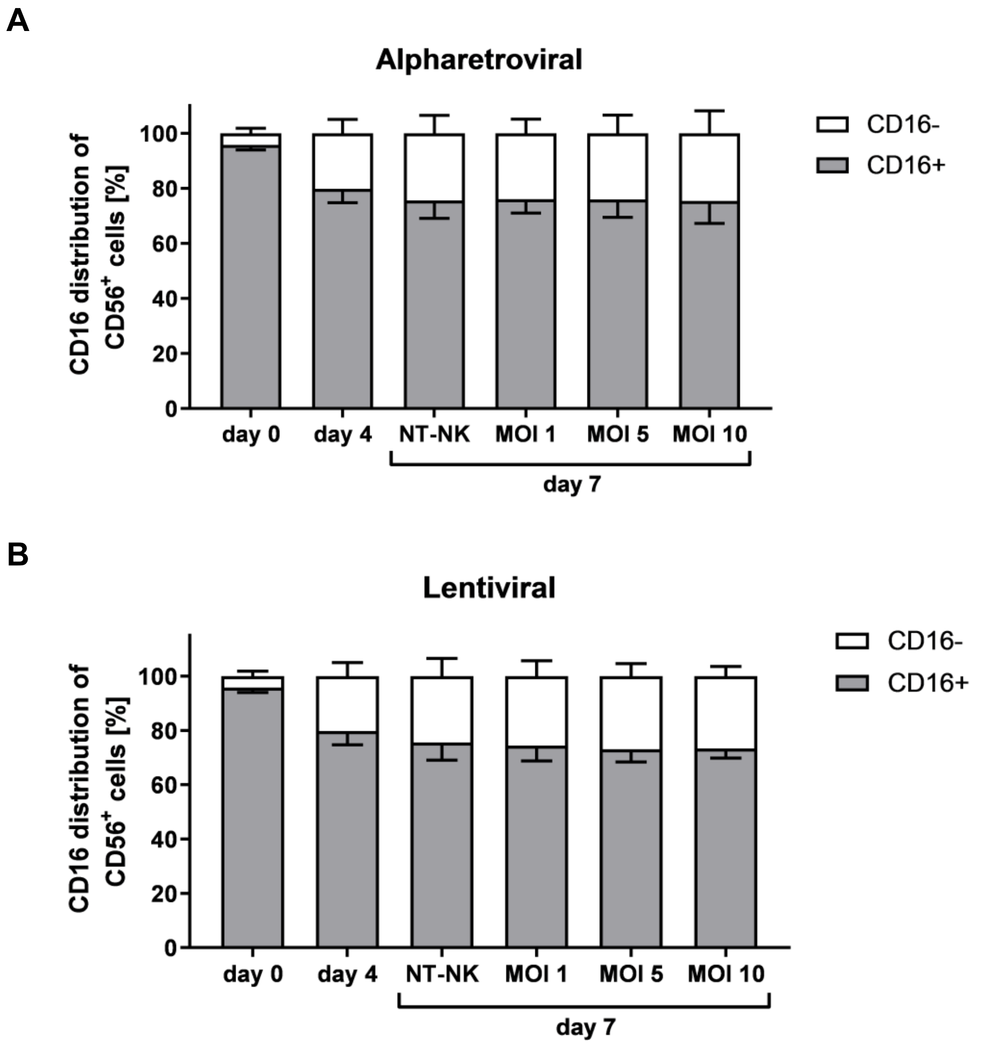

Supplementary Figure 8: CD16 expression of NK cells transduced with the alpharetroviral/RD114-TR or lentiviral/VSV-G CD19-CAR using Vectofusin-1 as shown in Figure 3C.

CD16 expression of NK cells ($CD56^{+}CD3^{-}$) was determined on day of isolation (day 0), prior transduction (day 4) and on day 7, as transduction efficiency was analyzed. **(A)** shows CD16 expression of NK cells isolated by NK cell enrichment and transduced on day 4 using Vectofusin-1 and RD114-TR pseudotyped alpharetroviral CD19-CAR particles. **(B)** shows CD16 expression of NK cells isolated by NK cell enrichment and transduced on day 4 using Vectofusin-1 and VSV-G pseudotyped lentiviral CD19-CAR particles. On day 7 NT-NK cells were analyzed as controls. NK cells in **(A)** and **(B)** were derived from four different donors *(n = 4)*. Shown are mean values ± SD.

A

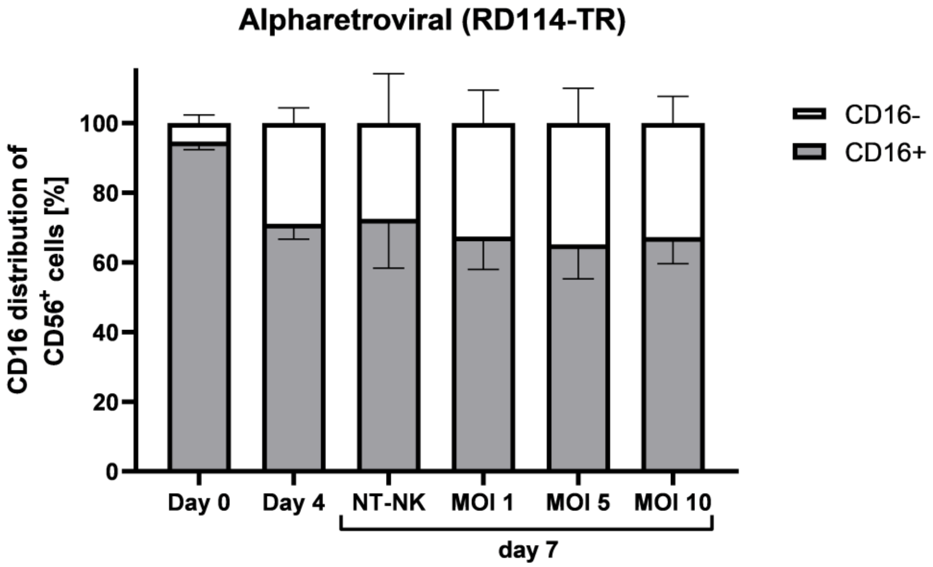

B

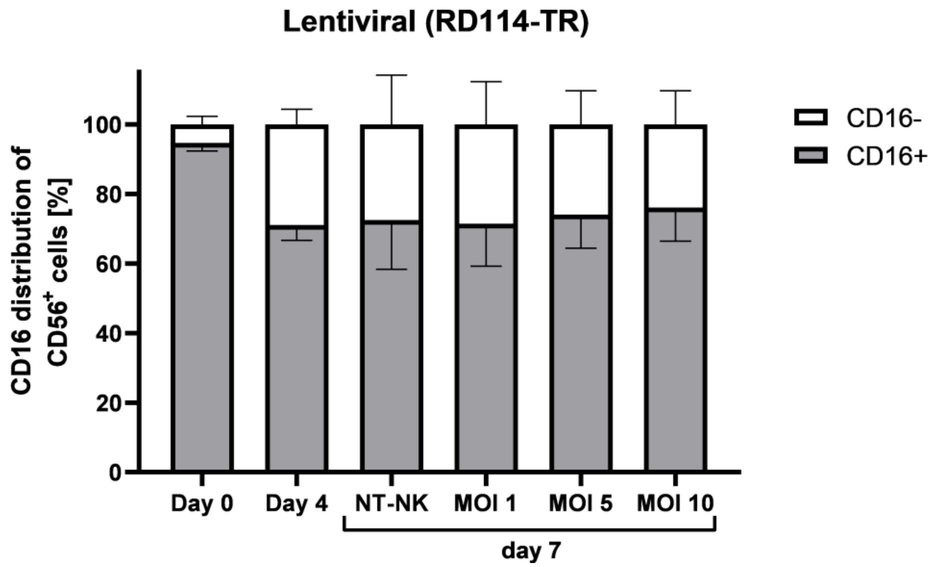

Supplementary Figure 9: CD16 expression of NK cells transduced with the alpharetroviral or lentiviral (RD114-TR) CD19-CAR using Vectofusin-1 as shown in Figure 6A.

CD16 expression of NK cells ($CD56^+CD3^-$) was determined on day of isolation (day 0), prior transduction (day 4) and on day 7, as transduction efficiency was analyzed. **(A)** shows CD16 expression of NK cells isolated by NK cell enrichment and transduced on day 4 using Vectofusin-1 and RD114-TR pseudotyped alpharetroviral CD19-CAR particles. **(B)** shows CD16 expression of NK cells isolated by NK cell enrichment and transduced on day 4 using Vectofusin-1 and RD114-TR pseudotyped lentiviral CD19-CAR particles. On day 7 NT-NK cells were analyzed as controls. NK cells in **(A)** and **(B)** were derived from four different donors *(n = 4)*. Shown are mean values ± SD.

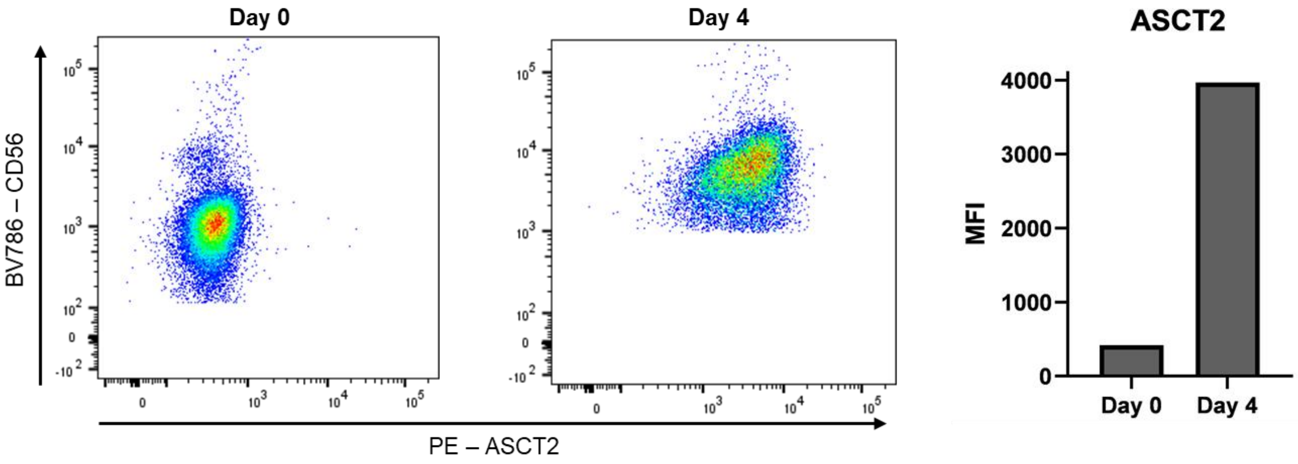

Supplementary Figure 10: ASCT2 expression of NK cells.

ASCT2 expression of NK cells was determined on day of isolation (day 0) and after a stimulation period of four days with IL-15 (day 4 – day of transduction). To determine ASCT2 expression of NK cells after surface staining with fluorochrome-conjugated antibodies against CD56 BV786 (clone NCAM16.2), CD3 BUV395 (clone Sk7) and CD16 PE-CF 594 (clone 3G8), an intracellular immunostaining was performed. Therefore, NK cells were fixed with formaldehyde for 15 minutes, washed and afterwards permeabilized with permeabilization buffer containing 0,2% Saponin (Fa.: Sigma) and 1% BSA (Sigma Aldrich) and simultaneously incubated with ASCT2 (V501) primary antibody (Cell Signaling Technologies) for one hour. Cells were then washed with PermWash-Buffer (BD Biosciences) before staining them with PE-conjugated secondary antibody anti-rabbit IgG (H+L), F(ab')2 Fragment (Cell Signaling Technologies) for 30 minutes. Finally, NK cells were analyzed by flow cytometry and gated as shown in Supplementary Figure 3. **(A)** shows ASCT2 expression of NK cells on day 0 and day 4. Shown dot plots match dot plot F in Supplementary Figure 3. **(B)** shows mean fluorescence intensities (MFI) of dot plots shown in **(A)**.

Supplementary References

1. Suerth JD, Maetzig T, Galla M, Baum C, Schambach A. Self-inactivating alpharetroviral vectors with a split-packaging design. *J Virol*. 2010;84(13):6626-6635. doi:10.1128/JVI.00182-10.
2. Oelsner S, Friede ME, Zhang C, et al. Continuously expanding CAR NK-92 cells display selective cytotoxicity against B-cell leukemia and lymphoma. *Cytotherapy*. 2017;19(2):235-249. doi:10.1016/j.jcyt.2016.10.009.
